# Emergence and Modular Evolution of a Novel Motility Machinery in Bacteria

**DOI:** 10.1371/journal.pgen.1002268

**Published:** 2011-09-08

**Authors:** Jennifer Luciano, Rym Agrebi, Anne Valérie Le Gall, Morgane Wartel, Francesca Fiegna, Adrien Ducret, Céline Brochier-Armanet, Tâm Mignot

**Affiliations:** 1 Institut de Microbiologie de la Méditerranée (IFR88)–Laboratoire de Chimie Bactérienne, CNRS UPR 9043, Marseille, France; 2 Aix-Marseille University, Marseille, France; Universidad de Sevilla, Spain

## Abstract

Bacteria glide across solid surfaces by mechanisms that have remained largely mysterious despite decades of research. In the deltaproteobacterium *Myxococcus xanthus*, this locomotion allows the formation stress-resistant fruiting bodies where sporulation takes place. However, despite the large number of genes identified as important for gliding, no specific machinery has been identified so far, hampering in-depth investigations. Based on the premise that components of the gliding machinery must have co-evolved and encode both envelope-spanning proteins and a molecular motor, we re-annotated known gliding motility genes and examined their taxonomic distribution, genomic localization, and phylogeny. We successfully delineated three functionally related genetic clusters, which we proved experimentally carry genes encoding the basal gliding machinery in *M. xanthus*, using genetic and localization techniques. For the first time, this study identifies structural gliding motility genes in the Myxobacteria and opens new perspectives to study the motility mechanism. Furthermore, phylogenomics provide insight into how this machinery emerged from an ancestral conserved core of genes of unknown function that evolved to gliding by the recruitment of functional modules in Myxococcales. Surprisingly, this motility machinery appears to be highly related to a sporulation system, underscoring unsuspected common mechanisms in these apparently distinct morphogenic phenomena.

## Author Summary

Motility over solid surfaces (gliding) is an important bacterial mechanism that allows complex social behaviours and pathogenesis. Conflicting models have been suggested to explain this locomotion in the deltaproteobacterium *Myxococcus xanthus*: propulsion by polymer secretion at the rear of the cells as opposed to energized nano-machines distributed along the cell body. However, in absence of characterized molecular machinery, the exact mechanism of gliding could not be resolved despite several decades of research. In this study, using a combination of experimental and computational approaches, we showed for the first time that the motility machinery is composed of large macromolecular assemblies periodically distributed along the cell envelope. Furthermore, the data suggest that the motility machinery derived from an ancient gene cluster also found in several non-gliding bacterial lineages. Intriguingly, we find that most of the components of the gliding machinery are closely related to a sporulation system, suggesting unsuspected links between these two apparently distinct biological processes. Our findings now pave the way for the first molecular studies of a long mysterious motility mechanism.

## Introduction

In Gram-negative bacteria, envelope machineries connecting the cell interior to the extracellular milieu must span all envelope layers, including the inner membrane, peptidoglycan and outer membrane. Despite these constraints, gram-negative bacteria have evolved sophisticated envelope nano-machines to interact with their environment. Conspicuous examples are bacterial organelles such as flagella, pili, and transport and secretion systems [1], [2]. In general, the structural genes encoding these systems are clustered within large transcriptional units allowing co-regulation of their expression. However, assembly also relies on additional complexity and must involve “just-in time” transcriptional regulations, specific targeting and protein self-assembly properties [3]. This raises the question of the evolutionary processes that led to the emergence of these macromolecular systems [4].

Non-homologous envelope macro-molecular structures mediate motility in bacteria. For example, bacteria swim in extremely viscous environments by means of a rotary flagellum, one of the most sophisticated known biological nano-machines [3]. Bacteria can also crawl across surfaces, for example, polymerization and de-polymerization of pilin fibers from the bacterial cell pole pull the cell forward, a “twitching” motility mechanism which also involves the coordinated assembly of many envelope proteins [5]–[7]. However, gram-negative rod-shaped bacteria are also able to move on surfaces by other means. For example, many bacteria move smoothly along their long axis in the absence of obvious extra-cellular organelles [8]. This gliding motility is associated with unusual flexibility of the cell body and can be found in very diverse bacterial phyla, such as, Bacteroidetes, Cyanobacteria and Deltaproteobacteria [8], [9]. In most species, the mechanism that drives gliding motility remains speculative. For example, in *Flavobacterium johnsoniae* (Bacteroidetes) gliding motility may be associated with a novel secretion apparatus. However, it is unclear whether this system is involved in assembly of the gliding machinery or constitutes the machinery itself [10]. Finally, gliding may be propelled differently in various species [8].

Despite decades of research, dedicated gliding motility machineries have not been identified unambiguously in any bacterial species, hampering detailed mechanistic studies and asking the question of the emergence of this process in bacteria. In *Myxococcus xanthus*, a gram negative deltaproteobacterium, surface motility allows the directed aggregation of thousands of cells into mounds that mature into fruiting bodies where the bacteria differentiate into spores [11]. *Myxococcus* cells can move by twitching motility, but in the absence of pili, the cells are still able to move, unmasking the activity of the gliding engine [11]. Recent cytological work suggested that motility is driven by protein complexes (Focal Adhesion Complexes, FAC) that push against the substratum as they accumulate periodically on the ventral side of the cell [12]–[14]. In a live cell assay, FACs can be observed as bright fluorescent fixed spots in cells expressing a fluorescent gliding motility protein (AglZ-YFP, [12]). The formation of AglZ-YFP foci requires the bacterial MreB-actin cytoskeleton [15] and a FACs-localized proton motive force-driven motor (AglRQS) was recently identified [13]. These observations suggest that AglRQS powers motility in concert with the MreB-cytoskeleton; however, how work from AglRQS is tranduced to the cell surface remains unknown and requires the identification of a motor-associated complex that spans the cell envelope.

In the past, 51 genes associated to defects in gliding motility were identified by transposon-based genetic screens, but the functional role of these genes in motility was not established [16], [17]. Recent work by Nan et al. [18] uncovered a new motility complex (AgmU, AglZ, AglT, AgmK, AgmX, AglW and CglB) and suggested that AgmU may be actively transported by PMF-utilizing motors [14]. However, the function of this complex and its direct link with the AglRQS motor remains to be established.

In this work, we aimed to identify the motility machinery conclusively. We re-investigated the 51 known *M. xanthus* gliding genes with the premise that the gliding machinery must have co-evolved with the AglRQS motor. This approach allowed us to identify a novel energy-driven protein complex, which we prove to be the basal gliding machinery. The results reveal the architecture of the gliding machinery and suggest a scenario of its emergence (and evolution) in bacteria.

## Results/Discussion

### Identification of candidate genes encoding the gliding machinery

Two independent transposon-based genetic screen studies [16], [17] identified 35 and 23 potential gliding motility genes, respectively (Table S1). Only seven genes overlapped in the two genetic studies, suggesting that the screens are not saturated and thus, the complete set of genes involved in gliding motility has likely not been identified. Nevertheless, these data constituted a good starting point and could indeed contain genes that encode the motility machinery. Irrespective of the exact motility mechanism, a number of cell envelope proteins should be part of the motility machinery. Therefore, we re-visited gene annotations specifically looking for genes encoding predicted membrane proteins, exported proteins and proteins containing motifs mediating protein-protein interaction, such as Tetratricopeptide repeat (TPR) and Coiled-coil domains (Table S1). A total of 28 genes were thus highlighted.

**Figure 1:**
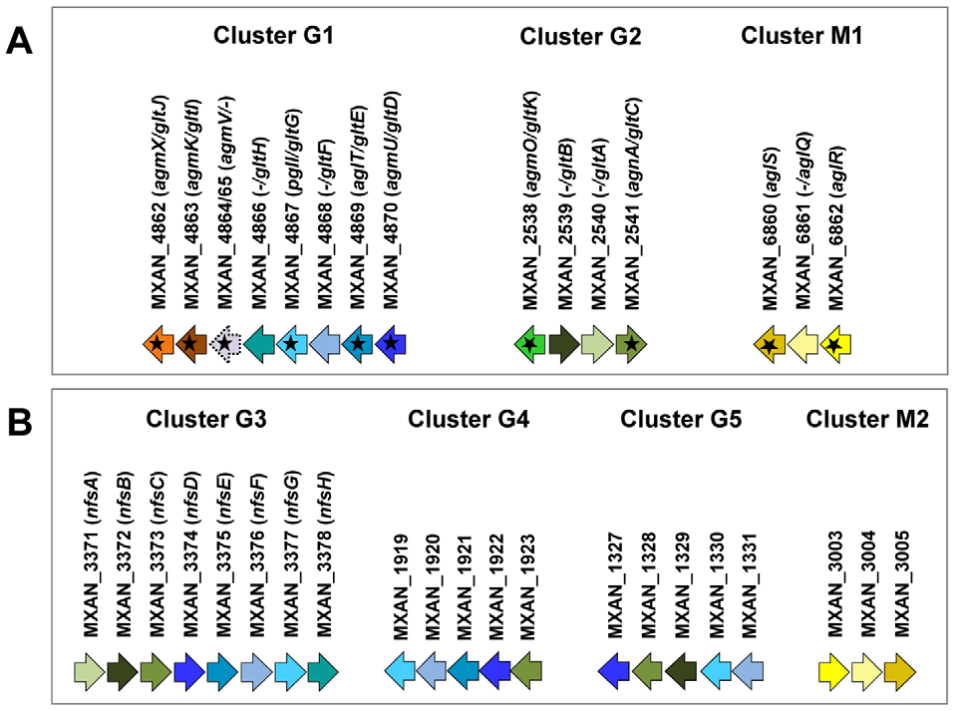
Genetic clusters carrying gliding motility genes in *M. xanthus*. (A) Genetic organisation of the 15 genes composing the G1, G2 and M1 clusters encoding the putative components of the gliding machinery in *Myxococcus xanthus* DK 1622. Predicted genes are indicated with their locus_tag, and their former and new names. The arrow that represents the putative MXAN_4864 and MXAN_4865 genes is a dotted line because they are likely pseudogenes (see text for more details). Stars indicate genes that were hit by the transposon screens [16], [17]. (B) Genetic organization of the G3, G4, G5 and M2 homologue clusters in *M. xanthus* DK 1622. The colour code indicates homologous genes and will be used throughout the study.

A careful survey of these 28 genes revealed that 13 gene hits were in fact clustered into four chromosomal regions of the *M. xanthus* DK 1622 genome. One region containing three hits, *aglW* (MXAN_5756, *tolB*), *aglX* (MXAN_5753, *tolQ*) and *aglV* (MXAN_5754, *tolR*), encoded together with MXAN_5755 (*tolA)* and MXAN_5757 (pal), *bona fide* components of a complete Tol-Pal system. Tol-Pal maintains envelope integrity and supports cell division in all bacteria where it has been studied [19], [20]. Thus, it is unlikely that Tol-Pal constitutes the motility machinery. Consistent with a general envelope function of the Myxococcus Tol-Pal, the *aglV* (*tolR*) mutant was also severely impaired in twitching motility [16]. We then focussed our analysis on the remaining three gene clusters (hereafter referred as Gliding1 (G1), Gliding 2 (G2) and Motor 1 (M1), Figure 1A). The G1 cluster contains eight genes, MXAN_4870-62, six of which have been hit by transposons: *agmU* (MXAN_4870), *aglT* (MXAN_4869), *pglI* (MXAN_4867), *agmV* (MXAN_4864/65, see below), *agmK* (MXAN_4863) and *agmX* (MXAN_4862) (Figure 1A). The G2 cluster contains four genes, MXAN_2538-41, two of them inactivated by transposons: *agmO* (MXAN_2538) and *agnA* (MXAN_2541). Finally, M1 contains the *aglRQS* genes themselves (MXAN_6862-60) and two hits by transposon insertions in *aglR* (MXAN_6862) and *aglS* (MXAN_6860). So overall, the G1, G2 and M1 clusters involve 15 genes, 10 of which have been previously hit by the transposon screens (Table 1).

**Table 1:** Bioinformatic analysis of G1, G2, and M1 cluster genes.

Cluster	Former name	New name	Locus-tag	Ref-seq	Length	Functional domain (Pfam)				Signal peptide	Transmembrane domain
						Name	Accession number	E-value	Position		
**Cluster G1**	*agmU*	*gltD*	MXAN_4870	YP_633028	1191	Tetratricopeptide repeat	PF07719	0.002	280-306	+	-
	*aglT*	*gltE*	MXAN_4869	YP_633027	471	Tetratricopeptide repeat	PF07719	5.7e-06	298–330	+	-
	*-*	*gltF*	MXAN_4868	YP_633026	89	-	-	-	-	+	-
	*pglI*	*gltG*	MXAN_4867	YP_633025	640	FHA domain Gram-negative bacterial tonB protein	PF00498PF03544	5.3e-131.8e-05	26–90570–637	-	+
	*-*	*gltH*	MXAN_4866	YP_633024	209	Autotransporter beta-domain	PF03797	0.084	73–153	+	+
	*agmK*	*gltI*	MXAN_4863	YP_633022	4132	Tetratricopeptide repeat Tetratricopeptide repeat	PF07719	8.7e-069.7e-05	3186–32183114–3142	-	-
	*agmX*	*gltJ*	MXAN_4862	YP_633021	674	-	-	-	-	-	+
**Cluster G2**	*agmO*	*gltK*	MXAN_2538	YP_630757	170	-	-	-	-	+	-
	*-*	*gltB*	MXAN_2539	YP_630758	275	-	-	-	-	+	-
	*-*	*gltA*	MXAN_2540	YP_630759	256	OmpA-like transmembrane domain	PF01389	0.011	168–255	+	-
	*agnA*	*gltC*	MXAN_2541	YP_630760	673	Tetratricopeptide repeat	PF07719	0.27*	260-289	+	-
**Cluster M1**	*aglR*	*-*	MXAN_6862	YP_634979	245	MotA/TolQ/ExbB proton channel family	PF01618	4.4e-18	112–217	-	+
	*-*	*aglQ*	MXAN_6861	YP_634978	162	Biopolymer transport protein ExbD/TolR	PF02472	6.7e-18	15–158	-	+
	*aglS*	*-*	MXAN_6860	YP_634977	194	Biopolymer transport protein ExbD/TolR	PF02472	3.1e-15	28–176	-	+

The M1 cluster encodes the component of a TolQR-like proton conducting motor, which has been characterized elsewhere [13]. The G1 and G2 cluster genes were analysed using public sequences and domain databases. The predicted MXAN_4866 (G1 region) and MXAN_2540 (G2 region) proteins are probably secreted and inserted in the outer membrane because they contain an Autotransporter ß-domain and adopt an OmpA-like fold, respectively (Table 1). AgmO may also be located in the outer-membrane because it carries a typical Outer-membrane Type-II signal sequence. TPR-repeats typically involved in multiprotein assemblies [21] are encoded by four G1 and G2 region genes: *agmU, aglT, agmK,* and *agnA* (Table 1). Among them, AgmU, AglT, and AgnA also carry signal peptides, suggesting that they are exported beyond the inner membrane. PglI (G1 region) is a predicted bi-topic transmembrane protein with a cytosolic ForkHead-Associated domain (FHA, [22]) and a periplasmic domain of unknown function. AgmX (G1 region) is also a potential integral membrane protein. MXAN_4868 and MXAN_2539 both carry N-terminal signal peptides but do not contain any conserved functional domains (Table 1). Finally, MXAN_4864 and MXAN_4865 are probably not actual genes and were discarded from this study (see Text S1 for justification) In the rest of this work, we tested if the G1 and G2 genes encode the AglRQS motor-associated gliding machinery. For clarity and to homogenize the nomenclature, we renamed all the G1 and G2 genes *glt* (**gl**iding **t**ransducer, see below), with *gltD, E, F, G, H, I* and *J* corresponding respectively to *agmU*, *aglT*, MXAN_4868, *pglI*, MXAN_4866, *agmK* and *agmX* (G1) and *gltC*, *A*, *B* and *K* corfresponding to *agnA*, MXAN_2540, MXAN_ 2539 and *agmO* (G2, see below and Text S2 for justification).

### The G1, G2, and M1 clusters may encode components of a single macro-molecular machinery

**Figure 2:**
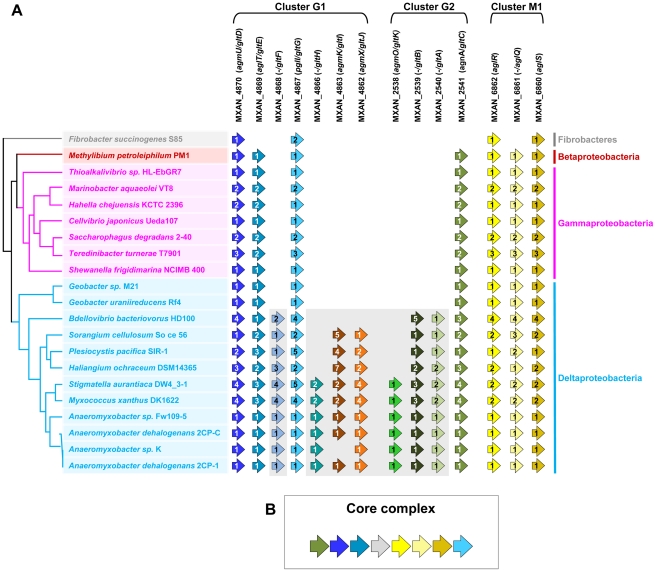
Taxonomic distribution of the closest homologues of the 14 genes composing the G1, G2, and M1 clusters, and genetic organization of the core complex. (A) For a given gene, the number of homologues in the corresponding genome is indicated by the numbers within arrows. The relationships between the species carrying the different homologues of the genes are indicated by the phylogeny on the left. Based on their taxonomic distribution, the 14 genes can be divided into Group A (grey background) and Group B (white background). (B) In all non Deltaproteobacteria and in *Geobacter*, the Group B genes clustered in a single genomic region.

The G1, G2 and M1 clusters encode a majority of potential envelope proteins and a motor complex (see above). A tempting hypothesis would be therefore that all these components constitute the gliding machinery. We systematically investigated the taxonomic distribution of the 14 genes defining the G1, G2 and M1 clusters in the 1180 complete prokaryotic proteomes available at the beginning of this study (see methods). The 14 genes could be separated in two distinct groups based on taxonomic distribution: A first group (Group A) contained seven genes (*gltF*, *gltH*, *gltI*, *gltJ*, *gltK*, *gltB* and *gltA*) that were only present (and sometimes in several copies) in Myxococcales (i.e. *Sorangium cellulosum, Plesiocystis pacifica, Haliangium ochraceum, Stigmatella aurantiaca, Myxococcus xanthus and the* four *Anaeromyxobacter sp.*) and in Bdellovibrionales (*Bdellovibrio bacteriovorus*) (Figure 2A). Such restricted taxonomic distribution suggested that these genes appeared only recently during the evolution of the Deltaproteobacteria. By contrast, a second group (Group B) contains seven genes with a much broader taxonomic distribution (Figure 2A). Specifically, blastp and PSI-BLAST queries identified 142 GltD, 2545 GltE, 313 GltG, 83 GltC, 2677 AglR, 2348 AglQ and 2385 AglS homologues. The taxonomic distributions of all these homologues are very different, suggesting that the corresponding genes have undergone different evolutionary histories, which was confirmed by preliminary phylogenetic analyses (not shown). However, in all these phylogenetic trees, the *M. xanthus* sequences emerge within a monophyletic clade containing homologues from other Deltaproteobacteria but also from a set of unrelated bacteria (i.e. one Betaproteobacteria, several Gammaproteobacteria and one member of Fibrobacteres, Figure S4). This strongly suggests that, although these genes belong to large gene families of distinct evolutionary histories, the *M. xanthus gltD*, *gltE*, *gltG*, *gltC*, *aglR*, *aglQ* and *aglS* genes and their closest homologues share a similar evolutionary history. The presence of Group B genes (sometime in several copies per genomes) in a few distantly related bacteria (Figure S1) suggests a complex evolutionary history punctuated with horizontal gene transfer (HGT) and gene duplication events (see below). In all non-Deltaproteobacteria and in *Geobacter*, Group B genes clustered in a single genomic region, possibly an operon, arguing strongly that they encode a single functional unit (i.e. core complex, Figure 2B and Figure S1, Tables S2 and S3). In all these bacteria, the core complex contains an additional gene that has no homologues in Myxococcales and Bdellovibrionales (Figure S1). Remarkably, group B genes (and thus the core complex) group genes from the G1 (*gltD*, *gltE* and *gltG*), G2 (*gltC*) and M1 (*aglQ, R and S*) clusters (Figure 2A-2B). This suggests an evolutionary link between the G1, G2 and M1 gene clusters. Strengthening this prediction, homologues of G1 and G2 clusters are grouped on the chromosome of the four *Anaeromyxobacter* relatives (Figure S1). Then, we proceeded to test the functional relationships between the G1, G2 and M1 genes.

### Genetic characterization of G1 and G2 gene clusters

In *M. xanthus*, many of the genes composing the G1 and G2 clusters were previously hit by genetic screens [16], [17]; however, the genes were only partially characterized, and it was not determined how they might be functionally related. More recently, Nan et al. [18] showed that individual deletions of the G1 genes *gltD-J* impair motility, but their analysis did not test whether these genes are structural. In fact, structural motility components cannot be simply discriminated from regulatory motility components solely based on mutational analysis and colony agar plate assays. First, the absence of motility at colony edges does not necessarily indicate that single cells are completely unable to move: for example, a class of directional mutants (FrzCD^c^, hyper active Frz-receptor mutants,[23]) forms smooth colony edges, yet, when observed under the microscope, individual cells glide but move back and forth at very high frequencies and thus show no net translocation (hyper-reversing cells, [23]). Thus, motility mutants must also be probed in single cell motility assays. Second, some mutations leading to complete motility defects can be suppressed by second-side mutations, showing that the mutated genes are not structural but regulatory. For example, the motility defect of the *aglZ* mutant is suppressed when *frz*, encoding a signal transduction system regulating the directionality of motility, is disrupted [24].

Thus, structural machinery genes must minimally meet the following criteria: (i) gene deletion should result in complete loss of motility in mutants that also lack twitching motility both at colony and single cell scales and (ii), the motility defect should not be suppressed by a *frz* mutation [24]. Consequently, in this study, we systematically combined the deletions to *pilA-* or *frzE*-null mutation (encoding the major pilin sub-unit and the essential FrzE kinase, respectively). It is still possible that regulatory genes may work independently from Frz, but, altogether, the genetic, localization and interaction evidence strongly supports that the Glt proteins are structural (see below).

We therefore made markerless in frame deletions in all the G1 and G2 genes (except *gltI* and *gltJ*) and showed that the deletions did not create polar effects by quantitative RT-PCR (Table 2 and Table 3). Of note, the expression of *gltH* was up-regulated 4-5 folds when *gltG* was deleted, which may point to a regulatory function of GltG (Table 2).

**Table 2:** *glt* mRNA expression in cluster G1 deletion strains.

Strain	Relevant genotype	Relative gene expression determined by q-RT-PCR				
		*gltD*	*gltE*	*gltF*	*gltG*	*gltH*
DZ2	Wild type	1	1	1	1	1
TM142	Δ*gltA*	ND^a^	0.63	0.40	0.77	0.65
TM148	Δ*gltB*	1.82	ND	1.89	1.24	1.15
TM136	Δ*gltC*	1.29	0.87	ND	1.24	0.72
TM135	Δ*gltD*	0.85	1.06	0.79	ND	4.66
TM149	Δ*gltE*	1.77	1.85	1.88	1.28	ND

a ND = Not Detected. The relative expression of the *gltD*, *gltE*, *gltF*, *gltG* and *gltH* genes in the wild-type strain and in deletion mutant strains was determined by q-RT-PCR. All the values are representative values from several independent experiments.

**Table 3:** *glt* mRNA expression in the cluster G2 deletion strains.

Strain	Relevant genotype	Relative gene expression determined by q-RT-PCR			
		*gltK*	*gltB*	*gltA*	*gltC*
DZ2	Wild type	1	1	1	1
TM142	Δ*gltK*	ND^a^	0,89	1,37	1,2
TM148	Δ*gltB*	1	ND	0,76	0,70
TM136	Δ*gltA*	0,99	0,54	ND	1,13
TM135	Δ*gltC*	0,59	0,70	0,61	ND

a ND = Not Detected. The relative expression of the *gltK*, *gltB*, *gltA* and *gltC* genes in the wild-type strain and in deletion mutant strains was determined by q-RT-PCR. All the values are representative values from several independent experiments.

**Figure 3:**
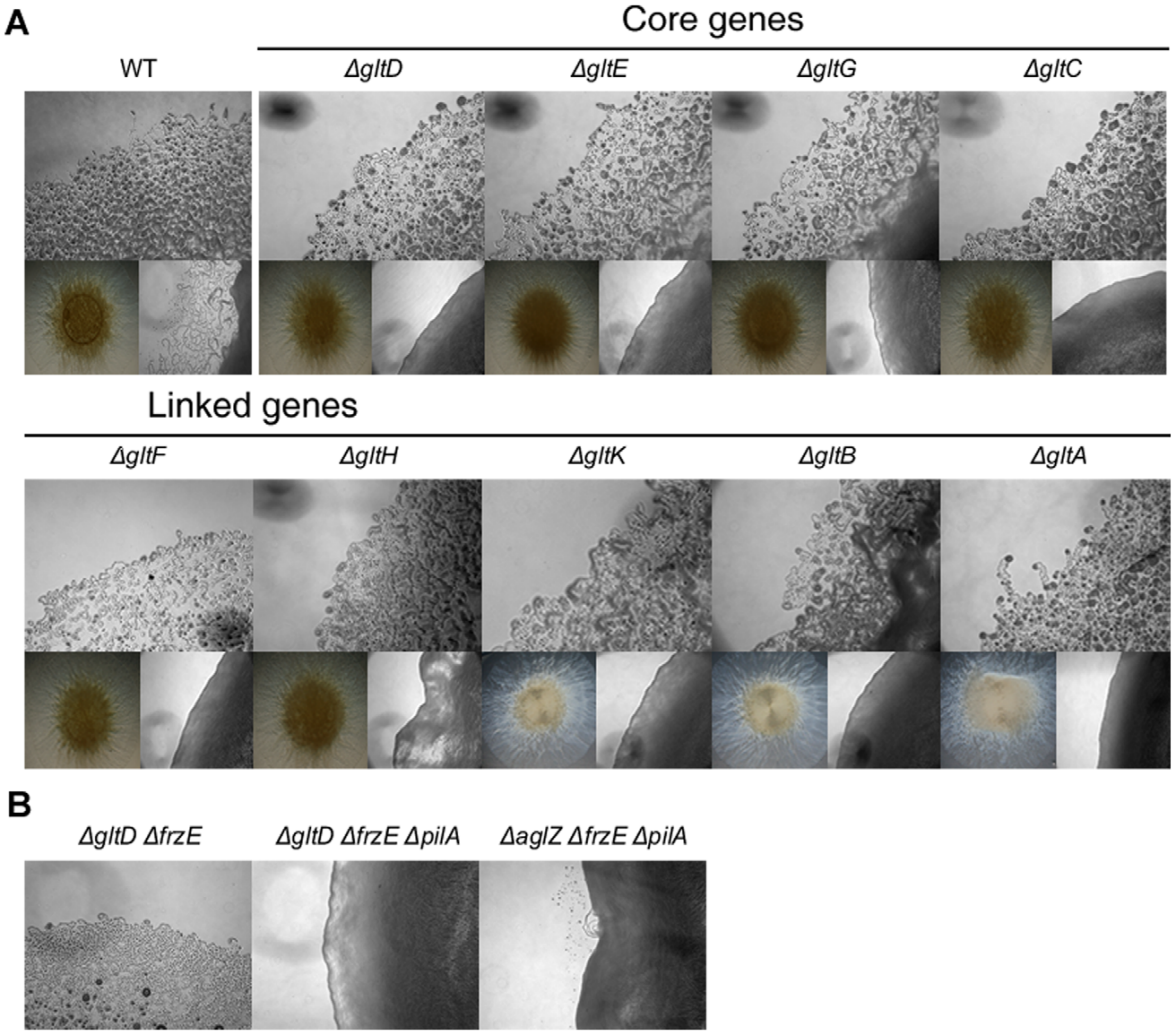
Group B genes encode structural components of the motility machinery. (A) Motility at the *gltA-H*and *gltK* deletion mutants colony edges after 48h incubation at 32°C on hard (1.5%) (upper panel). Lower left panel: twitching motility is unaffected in the *gltA-H* and *gltK* deletion mutants and observed in the form of expanded colony swarms on soft (0.5%). Lower right panel: motility of double *pilA gltA-H* and *pilA gltK* deletion mutants showing the complete absence of motility in these mutants. (B) Hard agar colony edges of the *gltD frzE, gltD frzE pilA* and *aglZ frzE pilA* mutants showing the lack of motility restoration in the *gltD* mutant. Note that single cells are clearly visible in the *aglZ frzE pilA* mutant, consistent with previous literature [24].

On agar plate assays, the *gltA-H and gltK* mutants retained intact twitching motility but were completely deficient in single cell motility at the colony edges (Figure 3A). *gltA-H* and *gltK pilA* double mutants were all completely non-motile both at the colony and single cell levels (Figure 3A and data not shown), showing that the *glt* genes are specific and essential to gliding motility. In one exception, the *gltH pilA* mutant showed small scales “jerky” displacements on occasions, but the motility defect was still very severe (Figure 3A and Video S1).

In a second step, we observed that *gltA-H* and *gltK frzE* double mutants were also completely non-motile in the colony and single cell assays (Figure 3B and data not shown). As a control, we also tested the simultaneous deletion of *frzE* and *aglZ* and observed that colony and single motility were both restored, as previously described (Figure 3B, [24]). Interestingly, group swarming appeared enhanced on hard agar plates in all cases, suggesting that the *frzE* mutation enhanced twitching in those mutants (Figure S2). Nan et al. [18] reported that motility of a *gltD* mutant allele was restored when a *frz* mutation was introduced, however this conclusion was based on observation of colony edges. In fact, enhanced twitching motility in the double mutant may have been mis-interpreted for restored gliding motility. To test this, we further introduced a *pilA* mutation in the double *gltD frzE* mutant. Motility was completely abolished in the resulting triple mutant. In contrast, the triple *aglZ frzE pilA* mutant was motile under similar conditions, as expected (Figure 3B, compare middle and right panels). The enhanced twitching in the *glt frzE* double mutants points to intriguing couplings between gliding and twitching motility, which will need further investigation.

In conclusion, the *glt* genes are genetically separable from *aglZ*, and may thus encode structural components of the motility machinery. A comparable genetic analysis also suggested that *aglR*, *Q* and *S* are structural [13]. Thus, the genetic results are consistent with a functional link between *aglRQS* and the *glt* G1 and G2 group genes.

### G1 cluster proteins localize to the cell envelope

**Figure 4:**
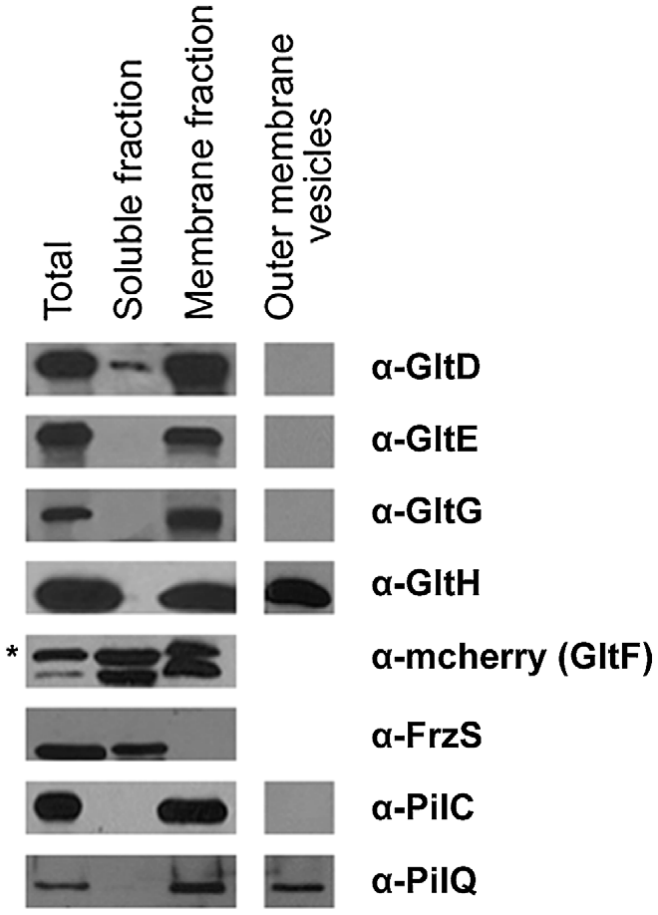
Envelope localization of the Glt proteins. Envelope localization of the Glt proteins. GltF localization is determined by western detection of the GltF-mCherry fusion (as indicated by the asterisk). FrzS, PilC and PilQ were used as control markers of the soluble, inner membrane and outer membrane fractions, respectively.

We next aimed to determine the subcellular localization of the suspected Glt protein complex. In absence of specific antibodies to detect all proteins, we only tested some proteins of the G1 cluster: GltD, E, F, G and H, all predicted to localize within the cell envelope (Table 1). We also tested the localization of a functional GltF-mCherry fusion with specific anti-mCherry antibodies. Cell fractionation experiments showed unambiguously that all five proteins localize in the cell envelopes (Figure 4). GltD was also present in the soluble fraction but to minor extents (Figure 4). GltF-mCherry was equally distributed in the soluble and membrane extracts (Figure 4). The GltF-mCherry fusion was functional (Figure S3 and data not shown), however it also seemed to be processed to some extent during the fractionation procedure (Figure 4), thus it cannot be excluded that its presence in the soluble fraction results from improper secretion.

We next wanted to discriminate inner- and outer-membrane proteins. Separating the inner membrane from the outer membrane was difficult using standard sucrose density gradients or detergent-based methods (see Methods). We therefore decided to harvest outer-membrane-derived vesicles (see Methods). Extracted vesicles contained PilQ, the pilus Secretin but not PilC, localizing at the inner membrane, confirming that the vesicles were derived from the outer membrane. Only GltH was detected in the vesicle preparation, which is consistent with the presence of an auto-transporter ß-domain in this protein (Figure 4, Table 1). All together these results suggest that GltD-G form an inner membrane localized complex that extends through the periplasm and connect the cell surface via the outer-membrane protein GltH.

### The Glt proteins form an AglRQS-associated dynamic motility complex

**Figure 5:**
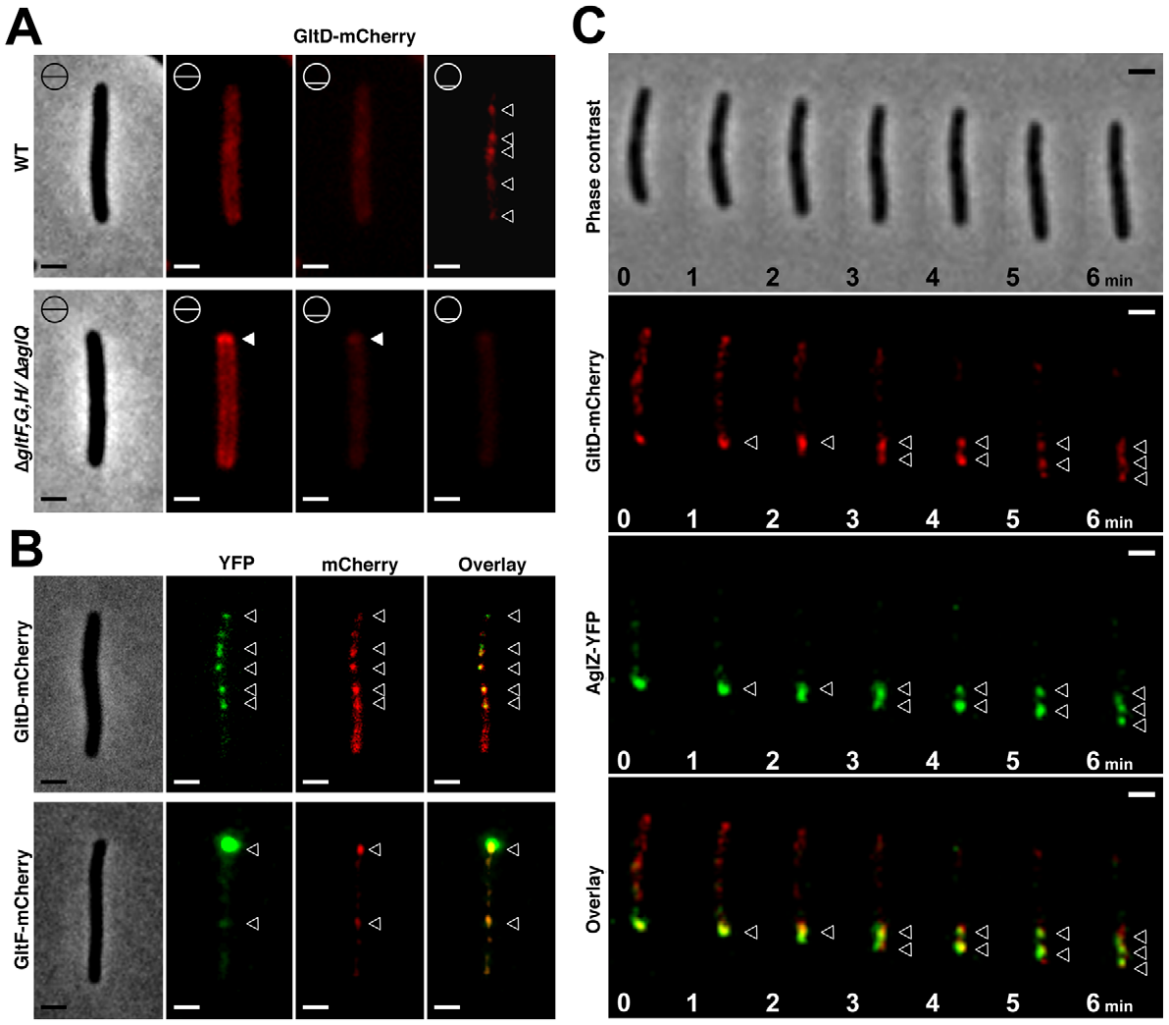
The Glt proteins localize dynamically to the AglZ-YFP clusters in a AglQ-dependent manner. (A) Localization of GltD-mCherry in different *z* sections in WT (upper panel) and mutant backgrounds (lower panel). Shown are unprocessed fluorescent micrographs of the different sections (position of the section along the *z* axis is indicated by a barred circle). Open triangles indicate GltD clusters. Scale bar  = 1 µm. (B) Co-localization of GltD- and GltF-mCherry with AglZ-YFP. Open triangles indicate clusters were the chimeric proteins co-localize. Scale bar  = 1 µm. (C) dynamic localization of GltD-mCherry and AglZ-YFP during movement. Scale bar  = 1 µm.

In a parallel study, we have demonstrated that the M1 cluster (*aglRQS*) encodes a proton-motive force-driven channel that produces motility traction forces at FACs [13]. The present study suggests that AglRQS and Glt proteins are functionally related, which needed to be proven experimentally. If the Glt proteins interact with the AglZ-AglRQS system, it would be expected that the Glt proteins also localize at FACs. A fluorescent functional GltD-mCherry fusion was already available [18]. We additionally obtained another functional fusion to GltF. In two other studies, GltD-mCherry was found to localize both in fixed clusters [18] and along a dynamic helix-like structure [14]. To rationalize this apparent dual localization pattern, it was proposed that GltD-mCherry molecules traffic along a helix and accumulate at FACs when they become engaged in propulsion [14]. In our hands, the pattern of GltD-, GltF-mCherry fluorescence in live cells was similar: fluorescence was mostly evident around the cell periphery; however, when we collected z-stacks of unprocessed images, fluorescent clusters became clearly apparent when the focal plane was focussed closer to the substratum (Figure 5A, 5B and Video S2). In moving cells, these clusters were fixed and largely co-localized with AglZ-YFP (Figure 5B and 5C). We were not able to resolve a helical pattern of GltD-mCherry around the cell periphery, but observing this structure may require mathematical image-deconvolution processing [14], which would explain this discrepancy. Nevertheless, these results show that GltD and GltF are recruited at FACs, and may be parts of a complex mediating contact between the exterior and the cell interior.

**Figure 6:**
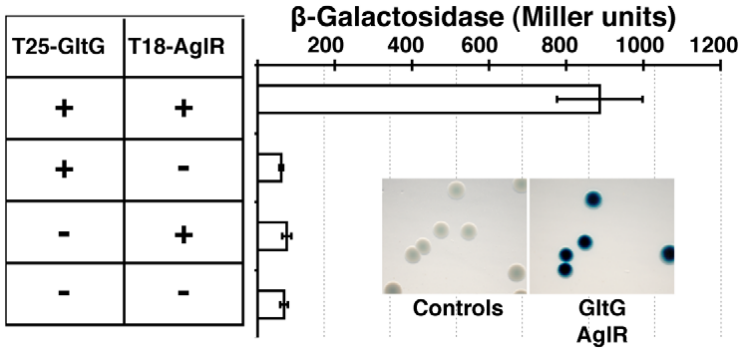
The AglRQS motor interacts directly with the gliding motility machinery. AglR interacts with GltG in a bacterial two-hybrid assay.

GltD-mCherry dynamics are dependent on the PMF [14], suggesting that they result from the activity of a motor, possibly the AglRQS complex (M1). Indeed, in an *aglQ* mutant, GltD- and GltF-mCherry failed to accumulate at FACs and were only localized around the cell periphery and at the cell poles (Figure 5A. and data not shown). To prove that AglRQS directly fuels trafficking of the Glt proteins, we searched which Glt protein may interact with the motor. By analogy to the Tol/Exb system, the AglR protein would deliver motor work to an output protein through an H^+^-driven conformational change in its N-terminal transmembrane helix [25]. Thus, the best candidate for direct interaction with AglR is the GltG protein. Indeed, this predicted transmembrane protein has a proline-rich TonB-like motif, typically found in TolA and TonB, the effector transducers in the Tol/Exb systems [20]. Moreover, GltG is the only predicted transmembrane protein that belongs to the core complex together with AglRQS (Figure 2B). We tested a potential interaction between AglR and GltG in a bacterial two-hybrid assay [26] (Figure 6). Highly significant β-galactosidase activity was only obtained when AglR and GltG were expressed together, showing that these proteins interact specifically (Figure 6). Finally, GltD-mCherry cluster localization was also abolished in mutants lacking *gltF, G* and *H*, further suggesting that these proteins are parts of one motility complex within the focal adhesion clusters (Figure 5A). All together, the results suggest that the AglRQS-Glt proteins assemble a dynamic envelope spanning motility machinery at the focal adhesion sites.

### Emergence and evolutionary history of the gliding machinery

Taken together, the computational and experimental results strongly suggest that the G1, G2 and M1 clusters contain genes encoding the major components of the gliding motility machinery. The most striking result of our *in silico* analysis is the discovery of a conserved core of genes (Group B) coding for several homologues of the gliding machinery components in non-gliding bacteria. To obtain further insights on the evolutionary mechanisms underlying the emergence of the gliding apparatus, we conducted an in-depth phylogenomic analysis (see Text S3). The phylogenies of the closest homologues of the seven genes defining the conserved core of genes (i.e. Group B) showed similar topologies (Figure S4). However, these analyses were based on a fairly small number of unambiguously aligned positions and as a result most of the nodes of the inferred trees were weakly supported (Bootstrap Values (BV) <90% and Posterior Probabilities (PP) <0.95, Figure S4). This caveat precluded the precise elucidation of the evolutionary histories of the components. To improve the resolution of the phylogenetic trees, we combined the group B genes *gltD, E* , *G* and *AglR,Q,S* in two distinct supermatrices (See Methods).

**Figure 7:**
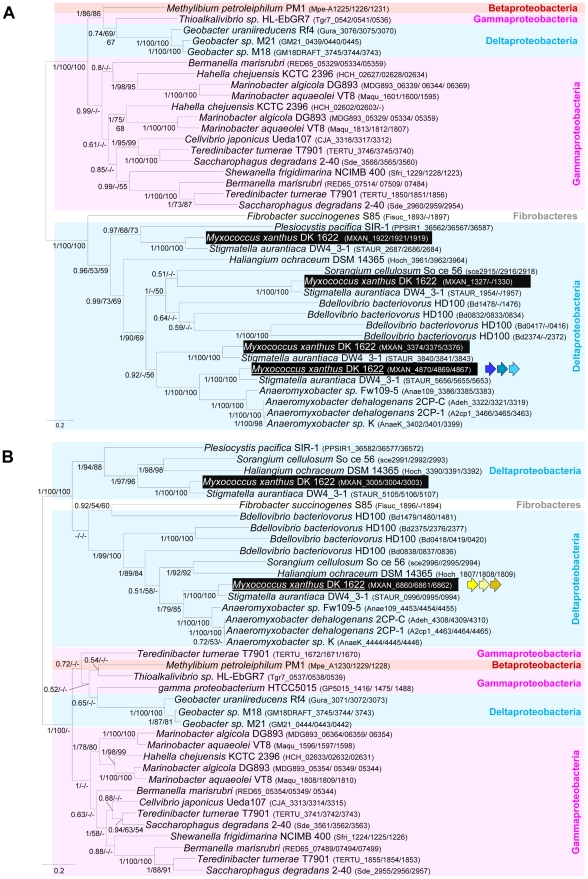
Co-evolution of *gltD-E-G* and *aglR-Q-S*. Rooted Bayesian phylogenetic trees of concatenated alignments of (A) GltD, GltE and GltG (39 sequences, 586 positions) and (B) AglR, AglQ and AglS (38 sequences, 376 positions). The root has been placed according to the phylogenies of the individual proteins. Numbers at nodes indicate posterior probabilities (PP) computed by MrBayes and bootstrap values (BV) computed by Treefinder and PhyML. Only PP and BV above 0.5 and 50% are shown. The scale bars represent the average number of substitutions per site. In each phylogenetic tree the putative *M. xanthus* gliding motility proteins are underlined and are illustrated with colour-coded gene symbols. For each species the individual locus_tags of the concatenated proteins are indicated in brackets. The position of multiple duplications of the concatenated proteins in *M. xanthus* are highlighted by black rectangles.

**Figure 8:**
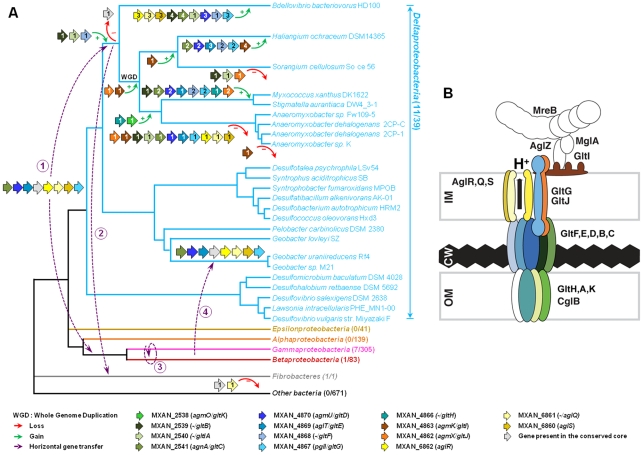
Evolution and structure of the *Myxococcus* gliding motility machinery. (A) Evolutionary scenario describing the emergence and evolution of the gliding motility machinery in *M. xanthus*. The relationships between organisms carrying close homologues of the 14 genes encoding putative components of the gliding machinery in *M. xanthus* are represented by the phylogeny. Green and red arrows respectively indicate gene acquisition and gene loss. The number of gene copies that were acquired or lost is indicated within arrows. The purple dotted arrows represent horizontal gene transfer events of one or several components. WGD marks the putative whole genome duplication event that occurred in the ancestor of Myxococcales. For each gene, locus_tag, former (*agm*/*agl*/*agn*) and new (*glt* and *agl*) names are provided. The number of complete genomes that contain homologues of *glt* and *agl* genes compared to the total number of complete genomes available at the beginning of this study are indicated in brackets. (B) The *Myxococcus* gliding machinery. The diagram compiles data from this work and published literature. Components were added based on bioinformatic predictions, mutagenesis, interaction and localization studies. Exhaustive information is not available for all proteins and thus the diagram largely is subject to modifications once more data will be available. Known interactions within the complex from experimental evidence are AglR-GltG, AglZ-MglA and interactions within the AglRQS molecular motor [13], [15]. For clarity, the proteins were colour-coded as in the rest of the manuscript.

As expected, the trees based on each supermatrix showed better resolutions than the individual gene trees (compare PP and BV in Figure 6 and Figure S4). Consistent with the single phylogenies (Figure S4), two separate clades (at odds with the species phylogeny) were observed in the resulting phylogenetic trees (PP = 1.00 and BV = 100%, Figure 7): More precisely, the three *Geobacter* representatives (Deltaproteobacteria) emerged within the Gammaproteobacteria, whereas *F. succinogenes* and the other Deltaproteobacteria, belonging to distinct phyla [27], emerged together in the *glt* and *agl* phylogenetic trees (Figure 7). Moreover, the relationships among the gammaproteobacterial sequences were mostly incongruent with the species phylogeny (Figure 2 and Figure 7). The discrepancy between the organism and gene trees precluded the clear identification of the precise bacterial lineage where the core complex originated, possibly the Gamma- or Deltaproteobacteria (Figure 8A). Nevertheless, HGT of the core complex is apparent: first, between Gammaproteobacteria and Deltaproteobacteria, then among Gammaproteobacteria and from Deltaproteobacteria to *Fibrobacter*, and last, from Gammaproteobacteria or Betaproteobacteria to *Geobacter* (Figure 8A, circles 1 to 4). In contrast, the restricted taxonomic distribution of the Group A genes indicates that they appeared and were recruited more recently during differentiation of the Deltaproteobacteria. An evolutionary scenario may thus be suggested: *gltA, B* and *F* likely appeared in the common ancestor of the Myxococcales and Bdellovibrionales, whereas *gltI* and *gltJ* (MXAN_4863-62) probably appeared in the ancestor of the Myxococcales, while *gltK and H* may have been acquired more recently (Figure 8A).

The evolutionary history of the genes involved in the gliding machinery is complicated by multiple duplication events, sometime followed by gene losses, which occurred in Myxococcales and Bdellovibrionales but also in Gammaproteobacteria (e.g. two *Marinobacter*, *Teredinibacter turnerae* and *Saccharophagus degradans*) and Fibrobacteres (Figure 8A and Figure S1). As a result, the gene clusters are sometimes present in several copies in the genomes of some species (*i.e.* the G3, G4, G5 and M2 clusters in *M. xanthus*, Figure 1B). Interestingly, none of these copies can substitute for the motility functions of the G1, G2 and M1 genes suggesting that duplications were associated with the emergence of novel functions (see below).

### The *Myxococcus* gliding machinery and its distribution in bacteria

The data strongly suggest that an ancestral cluster of genes containing GltD, E, G, C and AglR, Q, S (Group B genes) evolved into a motility machinery after sequential recruitment of new components, namely GltF, H, I, J, A B and K (Group A genes). Obviously, ancient genetic linkages were lost during the evolution of the gliding machinery, explaining why it has not been previously identified and precluding the rapid identification of all the motility genes. The Agl/Glt complex is likely the gliding machinery because: (i), individual mutations of all the *aglRQS* [13] and *glt* genes resulted in complete motility defects and were not suppressed by a second-site *frz* mutation. (ii), GltD- and GltF-mCherry fusions showed similar localization patterns and localized to fixed FACs like the AglRQS proteins [13] (iii) GltD localization depended on GltF, G and H and, (iv) AglR interacted with GltG in a bacterial two-hybrid study and the localization of GltD-mCherry depended on AglQ. We thus propose that mechanical work from the motor is transduced to the cell surface by the Glt complex through a specific interaction with GltG. Here, we have identified a minimal motility machinery gene set, and it cannot be excluded that more Glt proteins may emerge as functional and phylogenomic approaches will be continued. For example, interaction studies identified GltD, E, I, J to be part of a complex also containing CglB and AglW [18]. The functional relevance of these interactions still needs to be demonstrated. CglB is an outer membrane motility lipoprotein [28] that harbours a very restricted taxonomy distribution, being present only in Myxococcales genomes (except the four *Anaeromyxobacter*). CglB may be a surface-exposed components of the complex. However, AglW may not be specifically linked to motility because its genomic localization and its amino acid sequence indicate that it is a *bona fide* structural component of the Tol/Pal system (TolB).

The discovery of the Agl/Glt machinery now provides a new framework to elucidate the gliding motility mechanism (Figure 8B). A low-resolution architecture of the apparatus may be suggested by the genetic/localization/interaction and bioinformatic results (Figure 8B). AglRQS and GltG may constitute an inner membrane platform (this study) linked to the MreB cytoskeleton via proteins such as AglZ and MglA [15] on the cytosolic side and anchored to the substratum by a GltA-K complex in the cell envelope (Figure 8B). Nan et al. [14] proposed that the motility complex does not traverse the peptidoglycan layer but rather deforms it, generating transverse waves propagating down the axis of the cell. While this is plausible, the finding that GltH (and also potentially GltK and GltA based on bioinformatics predictions) is a critical outer-membrane component of the machinery rather argues that the motility complex is continuous through the cell envelope and contacts the cell exterior directly. More work will be required to understand the individual functions of the Glt proteins but the identification of the machinery gene set now opens investigations to understand the motility mechanism as a whole.

Elucidating the motility mechanism may be greatly facilitated by functional studies of the core complex (*aglR, Q, S* and *gltD, E, G, C*). The conservation and genetic linkage of these genes in gammaproteobacterial genomes suggest that they encode a functional protein complex of unknown function in these bacteria. It is unlikely that the core complex drives motility on its own because gliding is not documented in most bacteria where it is found and our study shows that the corresponding genes are not sufficient to drive motility. Based on this later observation, we propose that additional functional blocks (such as Group A genes) have been added sequentially to the original protein complex to convert it into a motility machinery (Figure 8A). What are these building blocks and how many of them remain to be discovered?

A recent study unambiguously showed that *Bdellovibrio bacteriovorus* is a *bona fide* glider [29]. While we cannot definitively rule out the independent emergence of gliding motility in this bacterium, we consider it unlikely: the *Bdellovibrio* genome contains four sets of expanded core complex suggesting that the Bdellovibrionales and Myxococcales gliding apparati are linked evolutionarily. Gliding motility may thus have emerged quite early in the ancestor of the Myxococcales and the Bdellovibrionales. The absence of homologues of GltH, I, J and K, all essential gliding proteins in *Myxococcus*, in the *Bdellovibrio* genome suggests that there are species specific requirements for gliding motility (Figure S1). *Bdellovibrio* cells are unusually small (less than 1 µm in length and 0.5 in diameter *vs* > µm in length and 1 µm in diameter for *Myxococcus*), which could explain some structural differences between gliding apparati. Based on the phylogenetic analysis of the *agl* components, the genes composing the Bd0828-0838 locus appear more closely related to *aglRQS* and may therefore constitute the best candidate to encode the *Bdellovibrio* gliding apparatus (Figure 7B). The Bd0828-0838 cluster also contains many homologues of *M. xanthus* gliding genes (with the exception of *gltH, I, J* and *K*, Figure 7A and Figure S1). Based on the *Bdellovibrio* example, it is tempting to speculate that any bacterium containing AglR,Q,S, GltD, E, F, G, C, A and B is a potential glider. This is for instance the case of *Myxococcus* close relatives, *Stigmatella aurantiaca* and the four *Anaeromyxobacter* species (Figure S1).

Finally, the *M. xanthus* gliding machinery is not conserved in bacteria belonging in other phyla (e.g. Bacteroidetes or Cyanobacteria), confirming that gliding motility evolved several times independently in Bacteria, as suggested by Jarrell and McBride [8].

### AglRQS/Glt-like machineries are exquisitely specialized

The presence of multiple copies of the G1, G2 and M1 clusters in Myxococcales (e.g. G3, G4, G5 and M2, in *M. xanthus* Figure 1B) likely results from duplication events. These duplications may be linked to the whole genome duplication event that occurred in the ancestor of the Myxococcales [30] and/or resulted from punctual gene-duplications during differentiation of the terminal branch of the Deltaproteobacteria. Duplications provide the raw material for the evolution of new gene functions [31], [32] and, for example, several regulation networks may have emerged this way in *Myxococcus* [30]. This study shows that the G3, G4, G5 and M2 clusters cannot compensate disruptions in the *glt* and *aglRQS* genes, already suggesting that they encode distinct functions. To further test this, we generated polar mutations in all the *gltD* homologues (MXAN_1922, G4; MXAN_1327, G5 and MXAN_3374, G3) and a deletion in MXAN_3004 (M2), the *aglQ* homologue [13]. None of these mutations impacted motility at any appreciable level, (Figure S5 and [13]). If the function of the G4, G5 and M2 regions is unknown, the G3 region was recently shown to be critical for sporulation and named *nfs* (necessary for sporulation, [33]). As expected, our *nfsD* (MXAN_3374) mutant failed to mature spores (data not shown). The *nfsA-H* genes are clustered in a single genomic region containing close homologues of G1 and G2 region genes (with the exception of GltI, J and K). Strikingly, the short evolutionary distances separating the *nfs* and *glt* genes in individual gene trees and in the *glt* supermatrix indicate that the *nfsA*-H genes are in fact the closest homologues of the *glt* genes (Figure 7 and Figure S4). Thus, the Glt and Nfs systems are a clear example of exquisite machinery specialization: in these cases, the ancestral core machinery has terminally differentiated to drive sporulation or gliding motility. In absence of more mechanistic insights, it is not clear which of the two processes is the most recent but this finding points to unsuspected similarities in these two distinct morphological processes. Comparative molecular analysis of the *nsf* and *glt* systems should be powerful to understand how these machineries function and how they can be specialized to enforce sporulation or gliding.

### Conclusions

In summary, the mechanism of gliding motility has remained mysterious despite three decades of research. A converging array of evidence now shows that motility is not propelled by slime secretion but results from PMF-energized trans-envelope complexes periodically distributed along the cell body (this study and [12], [13], [18]). However, how force is transduced from the AglRQS motor to the Glt proteins through the entire cell envelope and ultimately how that translates into motion, remains to be elucidated. The identification of the components of the gliding machinery now paves the way to address these questions. An immediate goal will be to characterize the motility machinery in an exhaustive manner, which we should be able to resolve combining bioinformatics, genetics and cell biology. In addition, the *M. xanthus* genome contains several gene clusters deriving from the ancestral core complex, but these copies are not functionally redundant and even specify non-motility related functions (*i.e.* the sporulation *nfs* system). Thus, Glt-like systems are remarkably linked to two fundamental processes of the *Myxococcus* life cycle and their acquisition may thus have been critical to the recent diversification of the Deltaproteobacteria. In the future, comparative analysis in *M. xanthus*, but also in the Delta and Gammaproteobacteria should be a powerful approach to elucidate the pathways that led to the evolution and diversification of complex bacterial envelope machineries.

## Materials and Methods

### Bacterial strains, plasmids, and growth

Primers and plasmids are listed in Tables S4 and S5. See Tables S6 and S7 for strains and their mode of construction. *M. xanthus* strains were grown at 32°C in CYE rich media as previously described [23]. Plasmids were introduced in *M. xanthus* by electroporation. Mutants and transformants were obtained by homologous recombination based on a previously reported method. Complementation of *gltG* and expression of GltF-mCherry were obtained after ectopic integration of the genes of interest at the Mx8-phage attachment site in appropriate deletion backgrounds (Table S6).

For phenotypic assays, cells (10 µl), at a concentration of 4×10^9^ cfu ml^−1^, were spotted on CYE plates containing agar concentrations of 0.5% or 1.5%, incubated at 32°C and photographed after 48 h with an Olympus SZ61 binocular or a Nikon Eclipse (model TE2000E) microscope (4x objective).

### mRNA extraction and QT-Reverse Transcription PCR

RNA from appropriate strains was extracted using a standard RNA purification kit (Promega). One microgram of total RNA was reverse-transcribed following the recommendations of the iScript cDNA Synthesis Kit (Bio-Rad). The resulting cDNA was then diluted (1/16), and 5 µl were used for the quantitative reverse transcription-PCR (q-RT-PCR) reaction. This step was performed on a Mastercycler ep realplex instrument (Eppendorf), using the SYBR Premix Ex Taq (Perfect Real Time) PCR kit (Takara Bio Group, Japan) according to manufacturer instructions in a final volume of 20 µl. Specific primers used for the reactions are described in Table S4. Melting curves were systematically analyzed to control for the specificity of the PCR reactions. The relative units were calculated from a standard curve plotting four different dilutions (1/80, 1/400, 1/2,000, and 1/10,000) against the PCR cycle number at which the measured fluorescence intensity reached the threshold (*C_T_*), corresponding to ∼10 times the standard deviation and thus significantly above the noise band of the baseline.

### Western blotting

Western blotting was performed as previously described [34] with 1/1000-1/5000 dilutions of polyclonal α-GltD, α-GltE, α-GltG, α-GltH (all raised for this study) and α-mCherry[13], α-PilC, α-PilQ [35] and α-FrzS [36].

### Preparation of cell membrane fractions and OMVs

Membrane Fractions and OMVs were purified from exponentially-growing cell cultures. Vegetative cells of *M. xanthus* were grown in CYE medium to an OD_600 nm_ = 0.7. For membrane fractions, cells were harvested by centrifugation at 8.000 rpm for 10 min at RT, resuspended in 50 mM Tris-HCl pH 7.6 and lysed by sonication. Cell debris were removed by low speed centrifugation (14000 rpm). The supernatants were then centrifuged at 45,000 *g* for 1 hr at 4°C. The resulting supernatants are enriched in soluble proteins. Pellets containing the crude envelope fractions (Inner and outer membrane) were resuspended in 50 mM Tris-HCl pH 7.6 and homogenized. The quality of the fractionation procedure was tested with antibodies to FrzS (soluble protein [36]) and PilC (inner membrane protein [35]).

Standard procedures to separate the inner membrane from the outer membrane using sucrose density gradients [37] did not successfully separate the two membranes. Detergent-based methods have been used successfully in *Myxococcus*, however in our case we could not prevent rapid degradation of the Glt proteins during the separation process [35]. OMVs are largely derived from the outer membranes, which was recently confirmed by proteomic analysis of the *Myxococcus* outer-membranes [38]. Thus, to test which Glt proteins are in the outer membranes we tested their presence in purified vesicules. For OMVs purification, cells and were discarded by centrifugation (8.000 rpm for 10 min at RT) and the culture supernatant was used for the isolation of vesicles. Culture supernatants (1 L) were passed through a 0.2 µm vacuum filter (Millipore). The resulting filtrate was centrifuged at 125 000× g for 2 h at 4°C to recover membrane vesicles. The supernatant was carefully removed and the vesicle pellet was resuspended in 50 mM Tris-HCl pH 7.6 and centrifuged at 180 000× g for 2 h at 4°C to concentrate and wash vesicles. The quality of the purification procedure was tested by electron microscopy (not shown) and antibodies to PilQ (outer membrane protein [35]) and PilC (inner membrane protein [35]).

### Bacterial two-hybrid experiments

Bacterial two-hybrid experiments, plate and ß-Galactosidase assays were performed as previously described [26] and as recommended by the manufacturer (Euromedex).

### Time lapse video-microscopy

Time lapse experiments were performed as previously described [39]. Microscopic analysis was performed using an automated and inverted epifluorescence microscope TE2000-E-PFS (Nikon, France). The microscope is equipped with “The Perfect Focus System” (PFS) that automatically maintains focus so that the point of interest within a specimen is always kept in sharp focus at all times, in spite of any mechanical or thermal perturbations. Images were recorded with a CoolSNAP HQ 2 (Roper Scientific, Roper Scientific SARL, France) and a 40x/0.75 DLL “Plan-Apochromat” or a 100x/1.4 DLL objective. All fluorescence images were acquired with appropriate filters with a minimal exposure time to minimize bleaching and phototoxicity effects.

Cell tracking was performed automatically using a previously described macro under the METAMORPH software (Molecular devices), when appropriate, manual measurements were also performed to correct tracking errors with tools built into the software. Typically, the images were equalized, straightened and overlaid under both ImageJ 1.40 g (National Institute of Health, USA) and METAMORPH.

### Annotation and mapping of gliding motility genes

The genetic screens of Youderian et al. ([16]) and Yu and Kaiser ([17]) allowed the identification of 35 and 23 potential gliding motility genes, respectively (Table S1). The function of the corresponding proteins was investigated using sequence similarity based approaches against the non-redundant (nr) database at the National Center for Biotechnology Information (NCBI, http://www.ncbi.nlm.nih.gov/) and Pfam (release 24.0) databases ([40]). The presence and location of signal peptide signal cleavage sites and of transmembrane helix were the predicted using the signalP 3.0 server ([41]) and TMHMM server v.2.0 ([42]). Finally, the location and the neighbourhood of each gene in the chromosome of *M. xanthus* DK 1622 were investigated using the complete genome sequence available at the NCBI ([30]; CP000113).

### Datasets construction

Homologues of each *M. xanthus* candidate protein were retrieved from a local database containing all complete prokaryotic proteomes available at the NCBI (April 8, 2010) using blastp with default parameters [43]. We also include in our analyses the genome sequences of *Stigmatella auriantiaca* DW4/3-1 and of *Plesiocystis pacifica* SIR-1 that came out in November 2010 and whose assembly is ongoing, respectively, both genomes being available at the NCBI. Importantly, the distinction between homologous and non-homologous sequences was assessed by visual inspection of each blastp outputs (no arbitrary cut-off on the E-value or score). To ensure the exhaustive sampling of homologues, iterative blastp queries were performed using homologues identified at each step as new seeds. PSI-BLAST queries were also used in order to recover very divergent homologues [43]. The absence of homologue in any complete proteome was systematically verified by tblastn queries against the nucleotide sequence of the corresponding genome. For each candidate protein, the retrieved homologues were gathered in a dataset. The corresponding sequences were aligned using the ClustalW2 program (Default parameters, [44]). Each alignment was visually inspected and manually refined when necessary using the ED program from the MUST package [45]. Regions where the homology between amino acid positions was doubtful were manually removed using NET from the MUST package.

Working on complete genomes may introduce major biases due to the taxonomic sampling of available complete genomes. Accordingly, for each candidate protein a second set of datasets based on homologues retrieved from the non-redundant (nr) protein database (the most exhaustive public database) at the NCBI was assembled. The taxonomic distribution and the phylogeny of homologues retrieved from either the nr database or from complete genomes showed similar patterns (data not shown). Thus, our analyses based on complete genomes are representative and reflect the taxonomic distribution of known homologues. Accordingly, only the results based on complete genomes will be presented in the results section.

The preliminary phylogenetic analyses of the candidate proteins allowed the identification of closest relatives of *M. xanthus* sequences. For each protein these homologues were gathered in a second dataset, the sequences were aligned and the resulting alignment was manually refined and cleaned like previously described. For the phylogenetic analyses of some of these datasets, we were removed some divergent sequences that can bias the phylogenetic reconstruction.

One approach to improve the resolution of the phylogenetic trees is to combine the genes that share a common evolutionary history in a single large alignment (also called supermatrix), [46]–[48]. Among the seven genes composing the Group B, *gltD, E* and *G* homologues are always clustered together in genomes and their individual phylogenies are very similar. Thus, these genes likely share a similar evolutionary history and can be used to build a supermatrix. For similar reasons, we combined the *aglR*, Q and S alignments in a second supermatrix. In contrast, *gltC* could neither be included in the *glt* nor in the *agl* supermatrix because it does not cluster physically with the corresponding genes in most Deltaproteobacteria. The Glt and Agl supermatrices were manually constructed by combining the cleaned alignments of GltDEG and AglQRS, respectively. When more than one homologue of these genes were present in a given genome, the genes were combined according to their physical linkage on the chromosome. For instance in the case of AglQRS, in *M. xanthus* the genes were combined as following: (i) MXAN_6860, _6861, _6862 and (ii) MXAN_3005, _3004, _3003.

### Phylogenetic analyses

For each individual and concatenated alignment, both Maximum likelihood (ML) and Bayesian phylogenetic trees were computed. ML analyses were run using PHYML version 3.0 with the Le and Gascuel (LG) model (amino acid frequencies estimated from the dataset) and a gamma distribution (4 discrete categories of sites and an estimated alpha parameter) to take into account evolutionary rate variations across sites [49]. The robustness of each branch was estimated by the non-parametric bootstrap procedure implemented in PhyML (100 replicates of the original dataset with the same parameters). Additional ML analyses were performed using TreeFinder with the same parameters [50]. Bayesian analyses were performed using MrBayes [51] with a mixed model of amino acid substitution including a gamma distribution (4 discrete categories) and an estimated proportion of invariant sites. MrBayes was run with four chains for 1 million generations and trees were sampled every 100 generations. To construct the consensus tree, the first 1500 trees were discarded as “burnin”.

## Supporting Information

Figure S1 Genetic organization of G1, G2, and M1 gene homologues in Deltaproteobacteria, Gammaproteobacteria, Betaproteobacteria and Fibrobacteres. The legend reads as in Figure 1. Dotted lines and question marks design putative highly diverging homologues that are proposed on the base of the genomic context surveys. Locus_tags are shown for all genes.
(PDF)

Figure S2 Enhanced twitching motility in the *ΩfrzE glt* mutants. Soft-agar colony assay showing *glt*-dependent de-repression of twitching motility in the *ΩfrzE* mutant. Scale bar  = 0.4 cm.
(PDF)

Figure S3 GltD-mCherry and GltF-mCherry are stably expressed. Western immunoblot using an anti-mCherry antiserum show stable expression of specific species of expected size. The arrows point to bands corresponding to the respective mCherry fusions.
(PDF)

Figure S4 Rooted Bayesian phylogenetic trees (A) of AgmU/GltD (MXAN_4870, 43 sequences, 379 positions), (B) of AglT/GltE (MXAN_4869, 37 sequences, 109 positions), (C) of PglI/GltG (MXAN_4867, 47 sequences, 78 positions), (D) of AgnA/GltC (MXAN_2541, 40 sequences, 185 positions), (E) of AglR (MXAN_6862, 38 sequences, 184 positions) and (F) of AglQ-AglS (MXAN_6861-6860, 76 sequences and 86 positions). The root has been placed accordingly to phylogenies based on whole gene families (not shown). Number at nodes indicates posterior probabilities (PP) and bootstrap support (BS) computed by Mrbayes and PhyMl, respectively. Only posterior probabilities and bootstrap values greater, respectively, than 0.5 and 50 % are shown. The scale bars represent the number of substitutions per site. In each phylogenetic tree proteins putatively involved in gliding in *M. xanthus* are underlined.
(PDF)

Figure S5 Motility phenotypes of the ΩMxan1327, ΩMxan1922 and Ω*nsfD*. Colony edges after 48 h incubation on hard (1,5%) agar show WT gliding motility. Insets: twitching motility on soft (0,5%) agar.
(PDF)

Table S1 List of the 23 and 35 Myxococcus xanthus genes identified by two transposon mutagenesis experiments (Yo  =  [16]; Yu  =  [17]). For each gene, the locus tag in the genome of *M. xanthus*, the accession number of the corresponding protein in the ref_seq database and the original functional annotation are provided. For each protein, we indicated the presence of functional conserved domains, of signal peptide and of transmembrane domains. $ signs design false positive genes that have been removed from the current version of the *M. xanthus* genome. Asterisks correspond to the 28 genes fitting our criteria as putative components of the gliding machinery. Among them we showed that nine (in red) co-localize in three small genomic regions.
(PDF)

Table S2 List of complete genomes of Deltaproteobacteria, Gammaproteobacteria, Betaproteobacteria and Fibrobacteres carrying homologues of the 14 candidates genes coding for the gliding machinery in *M. xanthus*. For each genome, the accession number in the nucleic ref_seq and in the GenBank databases, the size (in megabases) and the release date are provided.
(PDF)

Table S3 Exhaustive list of the homologues of the genes of the G1, G2 and M1 clusters found in complete genomes listed in Table S2. For each gene, the locus_tag, and the accession number, the length and the functional annotation of the corresponding proteins according to the ref_seq database are provided.
(PDF)

Table S4 Primers.
(PDF)

Table S5 Plasmids.
(PDF)

Table S6 *Myxococcus* strains.
(PDF)

Table S7 Plasmid constructions.
(PDF)

Text S1 MXAN4864 and MXAN4865 may not be actual motility genes.
(DOCX)

Text S2 Justification for a *glt* nomenclature of the gliding motility machinery genes.
(DOCX)

Text S3 Principle of the phylogenomic analysis.
(DOCX)

Video S1 “Jerky” motility phenotype of *gltH* mutant cells.
(MOV)

Video S2 Localization of GltD-mCherry in differents *z*-planes. Le bar within the circle indicates the positions of the respective focal planes relative to the short axis of the cell.
(AVI)
