# Supplementary material for: Emergence and Modular Evolution of a Novel Motility Machinery in Bacteria
Source: PLoS Genet. 2011 Sep 8;7(9):e1002268. doi: 10.1371/journal.pgen.1002268 (PMC3169522; doi:10.1371/journal.pgen.1002268)
Supplement: Figure S1 — Genetic organization of G1, G2, and M1 gene homologues in Deltaproteobacteria, Gammaproteobacteria, Betaproteobacteria and Fibrobacteres. The legend reads as in Figure 1. Dotted lines and question marks design putative highly diverging homologues that are proposed on the base of the genomic context surveys. Locus_tags are shown for all genes. (PDF) [file pgen.1002268.s001.pdf]

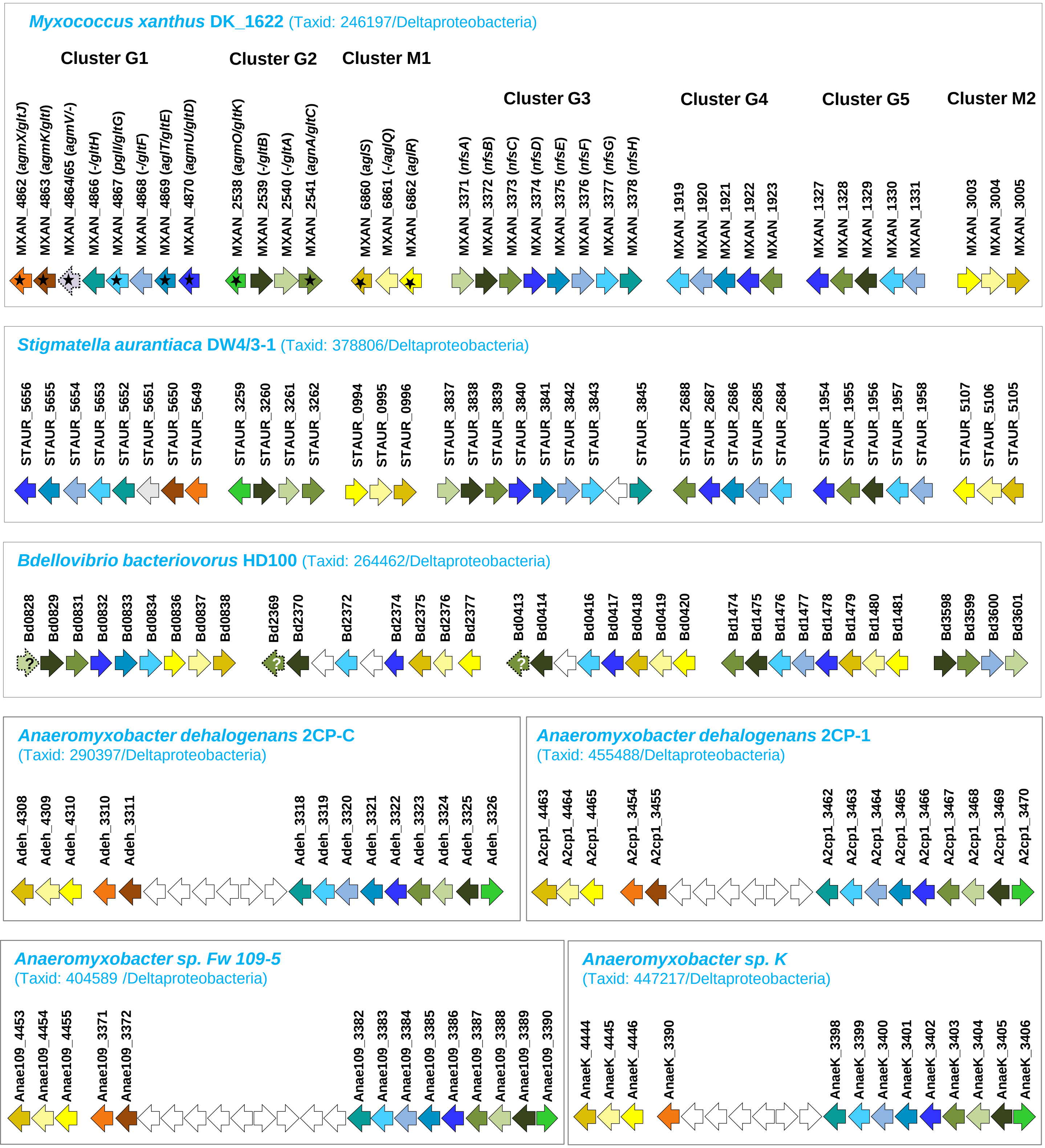
Myxococcus xanthus DK_1622 (Taxid: 246197/Deltaproteobacteria)
Cluster G1
Cluster G2
Cluster M1
Cluster G3
Cluster G4
Cluster G5
Cluster M2
MXAN_4862 (agmX/gltJ)
MXAN_4863 (agmK/gltI)
MXAN_4864/65 (agmV/-)
MXAN_4866 (-/gltH)
MXAN_4867 (pglI/gltG)
MXAN_4868 (-/gltF)
MXAN_4869 (aglT/gltE)
MXAN_4870 (agmU/gltD)
MXAN_2538 (agmO/gltK)
MXAN_2539 (-/gltB)
MXAN_2540 (-/gltA)
MXAN_2541 (agnA/gltC)
MXAN_6860 (aglS)
MXAN_6861 (-/aglQ)
MXAN_6862 (aglR)
MXAN_3371 (nfsA)
MXAN_3372 (nfsB)
MXAN_3373 (nfsC)
MXAN_3374 (nfsD)
MXAN_3375 (nfsE)
MXAN_3376 (nfsF)
MXAN_3377 (nfsG)
MXAN_3378 (nfsH)
MXAN_1919
MXAN_1920
MXAN_1921
MXAN_1922
MXAN_1923
MXAN_1327
MXAN_1328
MXAN_1329
MXAN_1330
MXAN_1331
MXAN_3003
MXAN_3004
MXAN_3005
Stigmatella aurantiaca DW4/3-1 (Taxid: 378806/Deltaproteobacteria)
STAUR_5656
STAUR_5655
STAUR_5654
STAUR_5653
STAUR_5652
STAUR_5651
STAUR_5650
STAUR_5649
STAUR_3259
STAUR_3260
STAUR_3261
STAUR_3262
STAUR_0994
STAUR_0995
STAUR_0996
STAUR_3837
STAUR_3838
STAUR_3839
STAUR_3840
STAUR_3841
STAUR_3842
STAUR_3843
STAUR_3845
STAUR_2688
STAUR_2687
STAUR_2686
STAUR_2685
STAUR_2684
STAUR_1954
STAUR_1955
STAUR_1956
STAUR_1957
STAUR_1958
STAUR_5107
STAUR_5106
STAUR_5105
Bdellovibrio bacteriovorus HD100 (Taxid: 264462/Deltaproteobacteria)
Bd0828
Bd0829
Bd0831
Bd0832
Bd0833
Bd0834
Bd0836
Bd0837
Bd0838
Bd2369
Bd2370
Bd2372
Bd2374
Bd2375
Bd2376
Bd2377
Bd0413
Bd0414
Bd0416
Bd0417
Bd0418
Bd0419
Bd0420
Bd1474
Bd1475
Bd1476
Bd1477
Bd1478
Bd1479
Bd1480
Bd1481
Bd3598
Bd3599
Bd3600
Bd3601
Anaeromyxobacter dehalogenans 2CP-C
(Taxid: 290397/Deltaproteobacteria)
Adeh_4308
Adeh_4309
Adeh_4310
Adeh_3310
Adeh_3311
Adeh_3318
Adeh_3319
Adeh_3320
Adeh_3321
Adeh_3322
Adeh_3323
Adeh_3324
Adeh_3325
Adeh_3326
Anaeromyxobacter dehalogenans 2CP-1
(Taxid: 455488/Deltaproteobacteria)
A2cp1_4463
A2cp1_4464
A2cp1_4465
A2cp1_3454
A2cp1_3455
A2cp1_3462
A2cp1_3463
A2cp1_3464
A2cp1_3465
A2cp1_3466
A2cp1_3467
A2cp1_3468
A2cp1_3469
A2cp1_3470
Anaeromyxobacter sp. Fw 109-5
(Taxid: 404589 /Deltaproteobacteria)
Anae109_4453
Anae109_4454
Anae109_4455
Anae109_3371
Anae109_3372
Anae109_3382
Anae109_3383
Anae109_3384
Anae109_3385
Anae109_3386
Anae109_3387
Anae109_3388
Anae109_3389
Anae109_3390
Anaeromyxobacter sp. K
(Taxid: 447217/Deltaproteobacteria)
AnaeK_4444
AnaeK_4445
AnaeK_4446
AnaeK_3390
AnaeK_3398
AnaeK_3399
AnaeK_3400
AnaeK_3401
AnaeK_3402
AnaeK_3403
AnaeK_3404
AnaeK_3405
AnaeK_3406

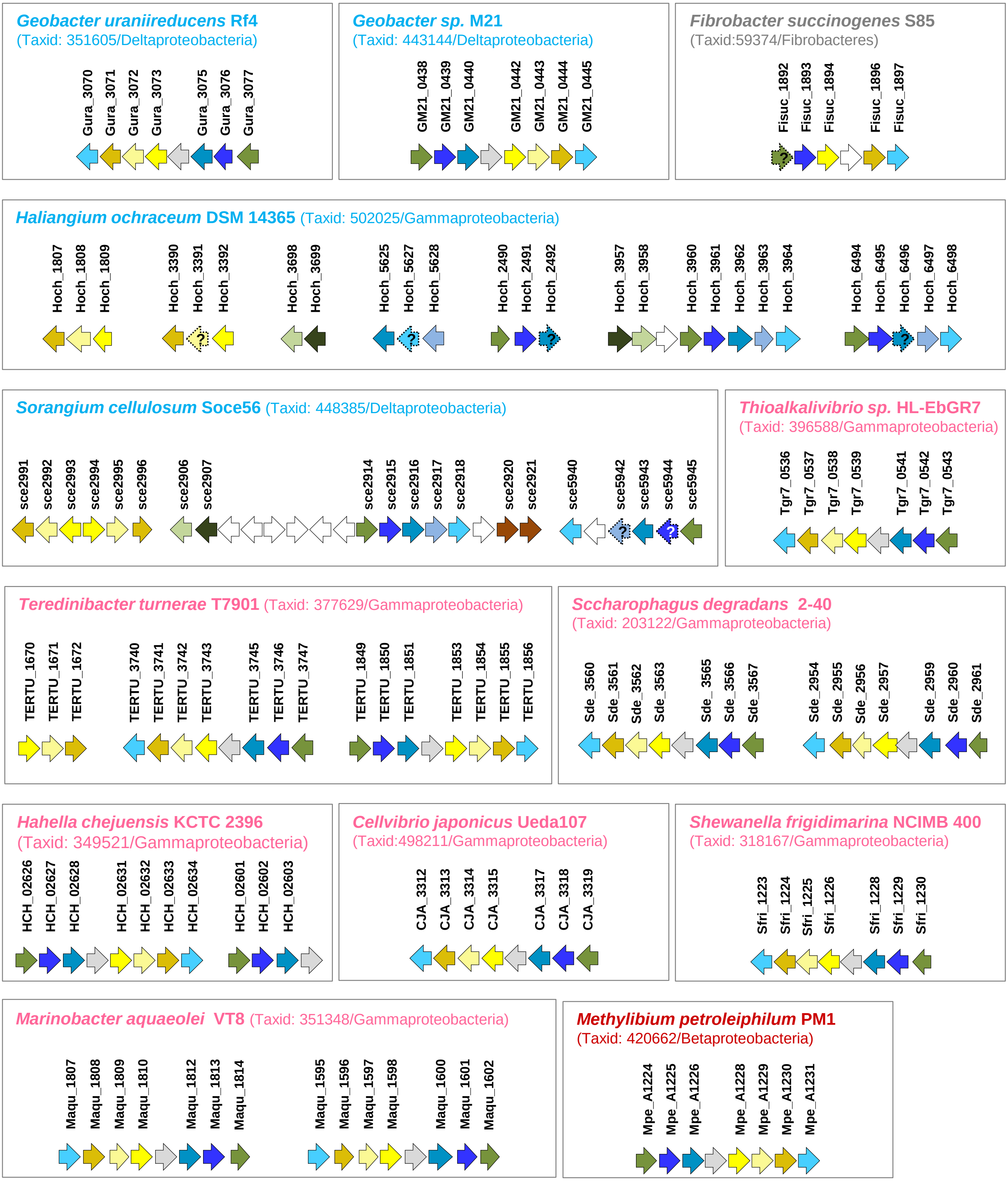
Geobacter uraniireducens Rf4
(Taxid: 351605/Deltaproteobacteria)
Gura_3070 Gura_3071 Gura_3072 Gura_3073 Gura_3075 Gura_3076 Gura_3077
Geobacter sp. M21
(Taxid: 443144/Deltaproteobacteria)
GM21_0438 GM21_0439 GM21_0440 GM21_0442 GM21_0443 GM21_0444 GM21_0445
Fibrobacter succinogenes S85
(Taxid:59374/Fibrobacteres)
Fisuc_1892 Fisuc_1893 Fisuc_1894 Fisuc_1896 Fisuc_1897
?
Haliangium ochraceum DSM 14365 (Taxid: 502025/Gammaproteobacteria)
Hoch_1807 Hoch_1808 Hoch_1809 Hoch_3390 Hoch_3391 Hoch_3392 Hoch_3698 Hoch_3699 Hoch_5625 Hoch_5627 Hoch_5628 Hoch_2490 Hoch_2491 Hoch_2492 Hoch_3957 Hoch_3958 Hoch_3960 Hoch_3961 Hoch_3962 Hoch_3963 Hoch_3964 Hoch_6494 Hoch_6495 Hoch_6496 Hoch_6497 Hoch_6498
?
Sorangium cellulosum Soce56 (Taxid: 448385/Deltaproteobacteria)
sce2991 sce2992 sce2993 sce2994 sce2995 sce2996 sce2906 sce2907 sce2914 sce2915 sce2916 sce2917 sce2918 sce2920 sce2921 sce5940 sce5942 sce5943 sce5944 sce5945
?
Thioalkalivibrio sp. HL-EbGR7
(Taxid: 396588/Gammaproteobacteria)
Tgr7_0536 Tgr7_0537 Tgr7_0538 Tgr7_0539 Tgr7_0541 Tgr7_0542 Tgr7_0543
Teredinibacter turnerae T7901 (Taxid: 377629/Gammaproteobacteria)
TERTU_1670 TERTU_1671 TERTU_1672 TERTU_3740 TERTU_3741 TERTU_3742 TERTU_3743 TERTU_3745 TERTU_3746 TERTU_3747 TERTU_1849 TERTU_1850 TERTU_1851 TERTU_1853 TERTU_1854 TERTU_1855 TERTU_1856
Sccharophagus degradans 2-40
(Taxid: 203122/Gammaproteobacteria)
Sde_3560 Sde_3561 Sde_3562 Sde_3563 Sde_3565 Sde_3566 Sde_3567 Sde_2954 Sde_2955 Sde_2956 Sde_2957 Sde_2959 Sde_2960 Sde_2961
Hahella chejuensis KCTC 2396
(Taxid: 349521/Gammaproteobacteria)
HCH_02626 HCH_02627 HCH_02628 HCH_02631 HCH_02632 HCH_02633 HCH_02634 HCH_02601 HCH_02602 HCH_02603
Cellvibrio japonicus Ueda107
(Taxid:498211/Gammaproteobacteria)
CJA_3312 CJA_3313 CJA_3314 CJA_3315 CJA_3317 CJA_3318 CJA_3319
Shewanella frigidimarina NCIMB 400
(Taxid: 318167/Gammaproteobacteria)
Sfri_1223 Sfri_1224 Sfri_1225 Sfri_1226 Sfri_1228 Sfri_1229 Sfri_1230
Marinobacter aquaeolei VT8 (Taxid: 351348/Gammaproteobacteria)
Maqu_1807 Maqu_1808 Maqu_1809 Maqu_1810 Maqu_1812 Maqu_1813 Maqu_1814 Maqu_1595 Maqu_1596 Maqu_1597 Maqu_1598 Maqu_1600 Maqu_1601 Maqu_1602
Methylibium petroleiphilum PM1
(Taxid: 420662/Betaproteobacteria)
Mpe_A1224 Mpe_A1225 Mpe_A1226 Mpe_A1228 Mpe_A1229 Mpe_A1230 Mpe_A1231
