# Supplementary figures and images for: Emergence and Modular Evolution of a Novel Motility Machinery in Bacteria
Source: PLoS Genet. 2011 Sep 8;7(9):e1002268. doi: 10.1371/journal.pgen.1002268 (PMC3169522; doi:10.1371/journal.pgen.1002268)

***ΩfrzE***

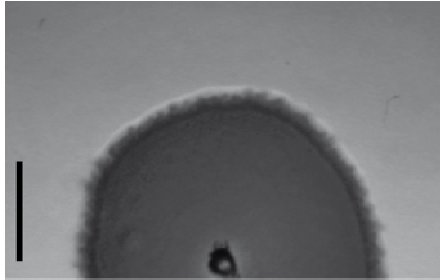

***ΩfrzE Δglt***

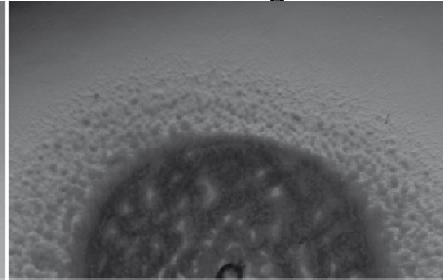

Supplement: Figure S2 — Enhanced twitching motility in the ΩfrzE glt mutants. Soft-agar colony assay showing glt-dependent de-repression of twitching motility in the ΩfrzE mutant. Scale bar = 0.4 cm. (PDF) [file pgen.1002268.s002.pdf]

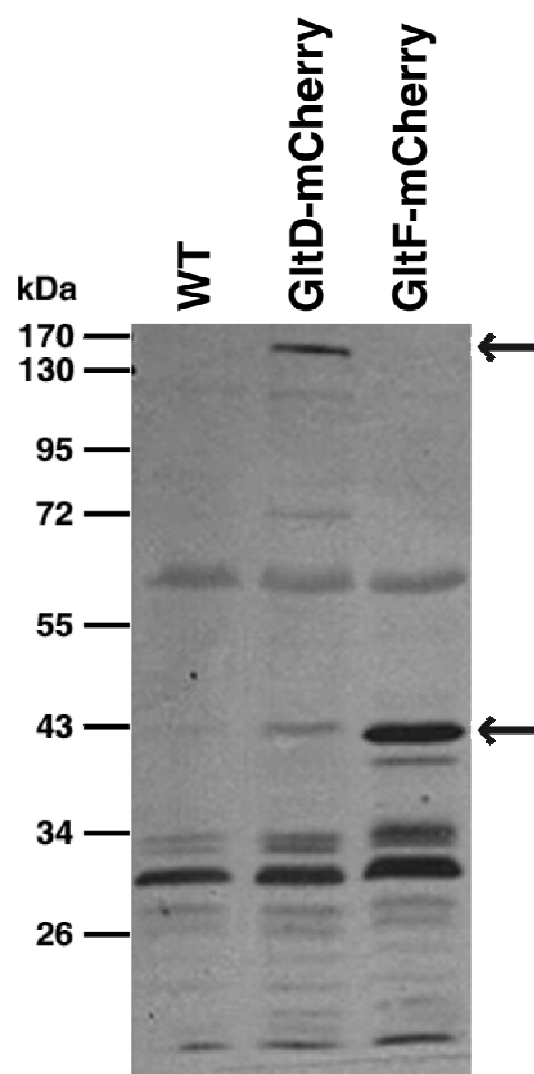

Supplement: Figure S3 — GltD-mCherry and GltF-mCherry are stably expressed. Western immunoblot using an anti-mCherry antiserum show stable expression of specific species of expected size. The arrows point to bands corresponding to the respective mCherry fusions. (PDF) [file pgen.1002268.s003.pdf]

A

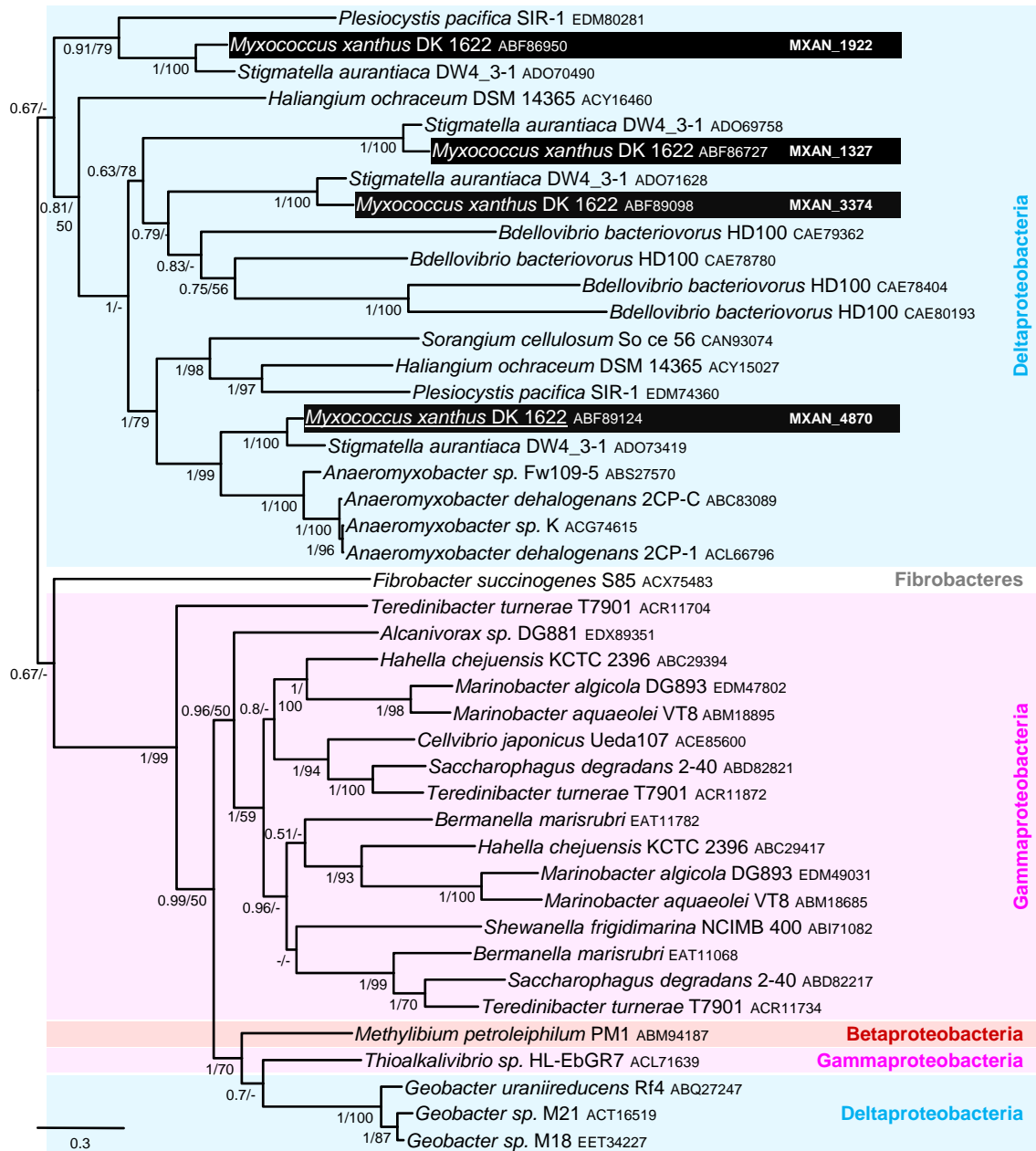

**B**

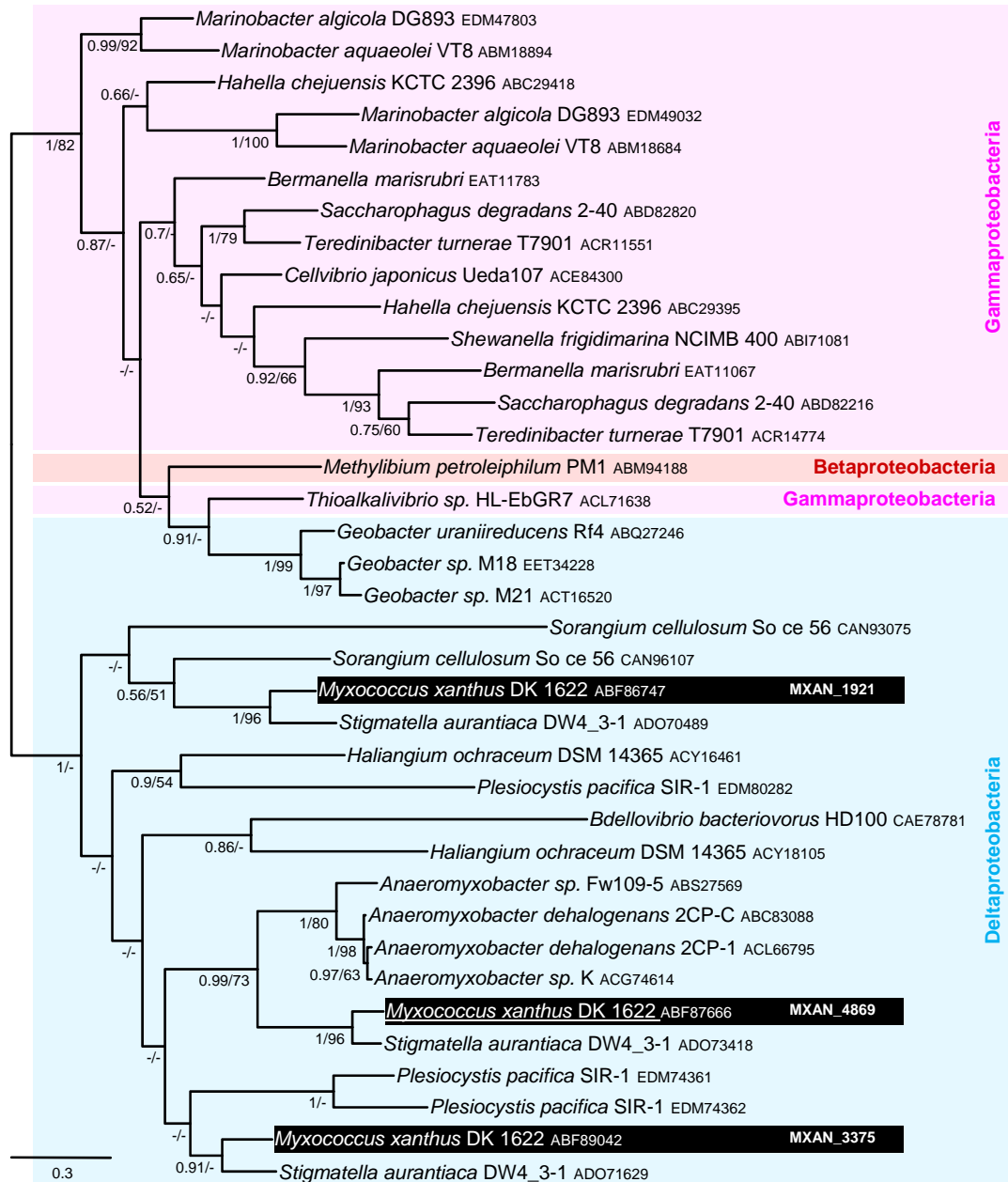

C

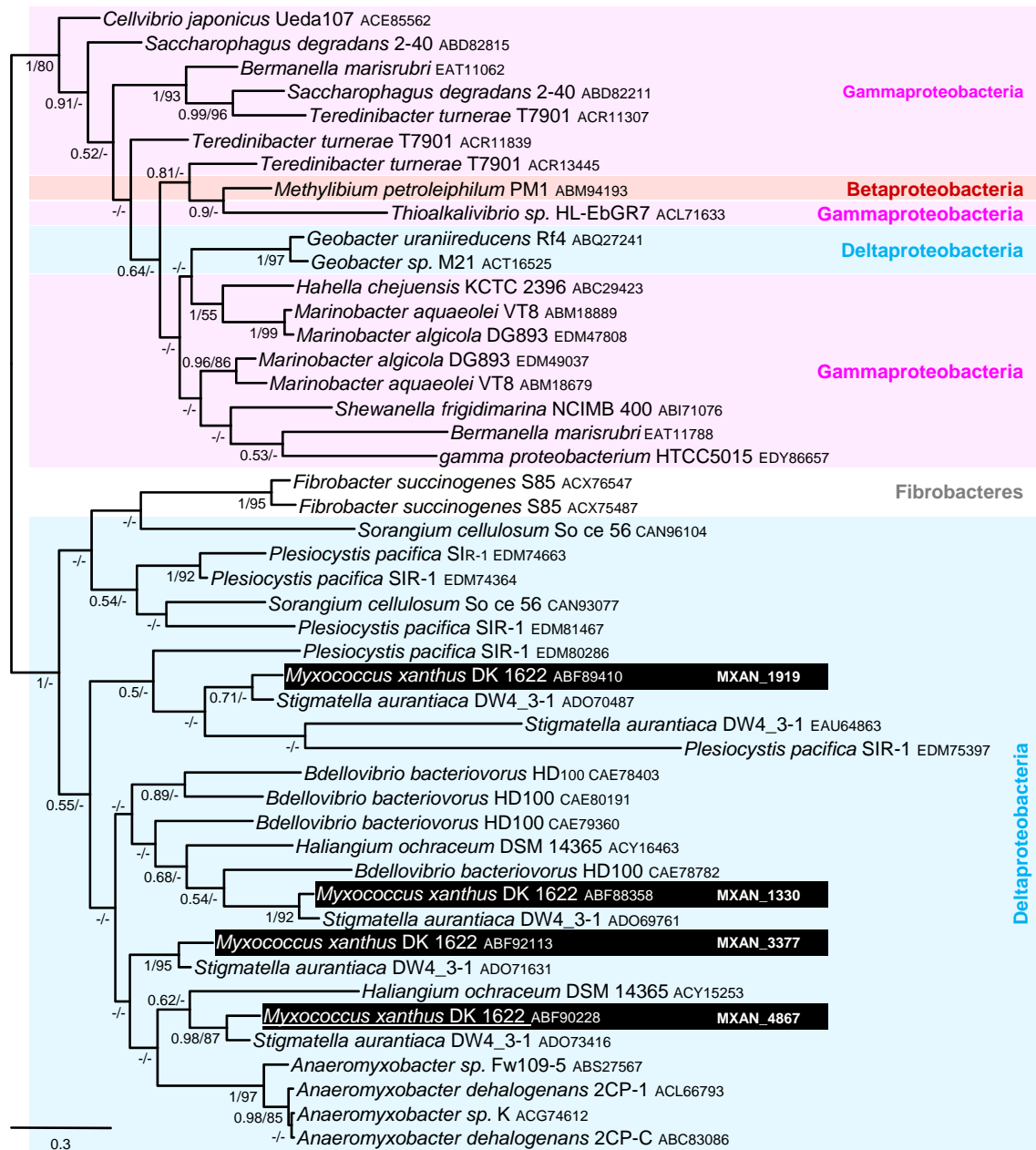

D

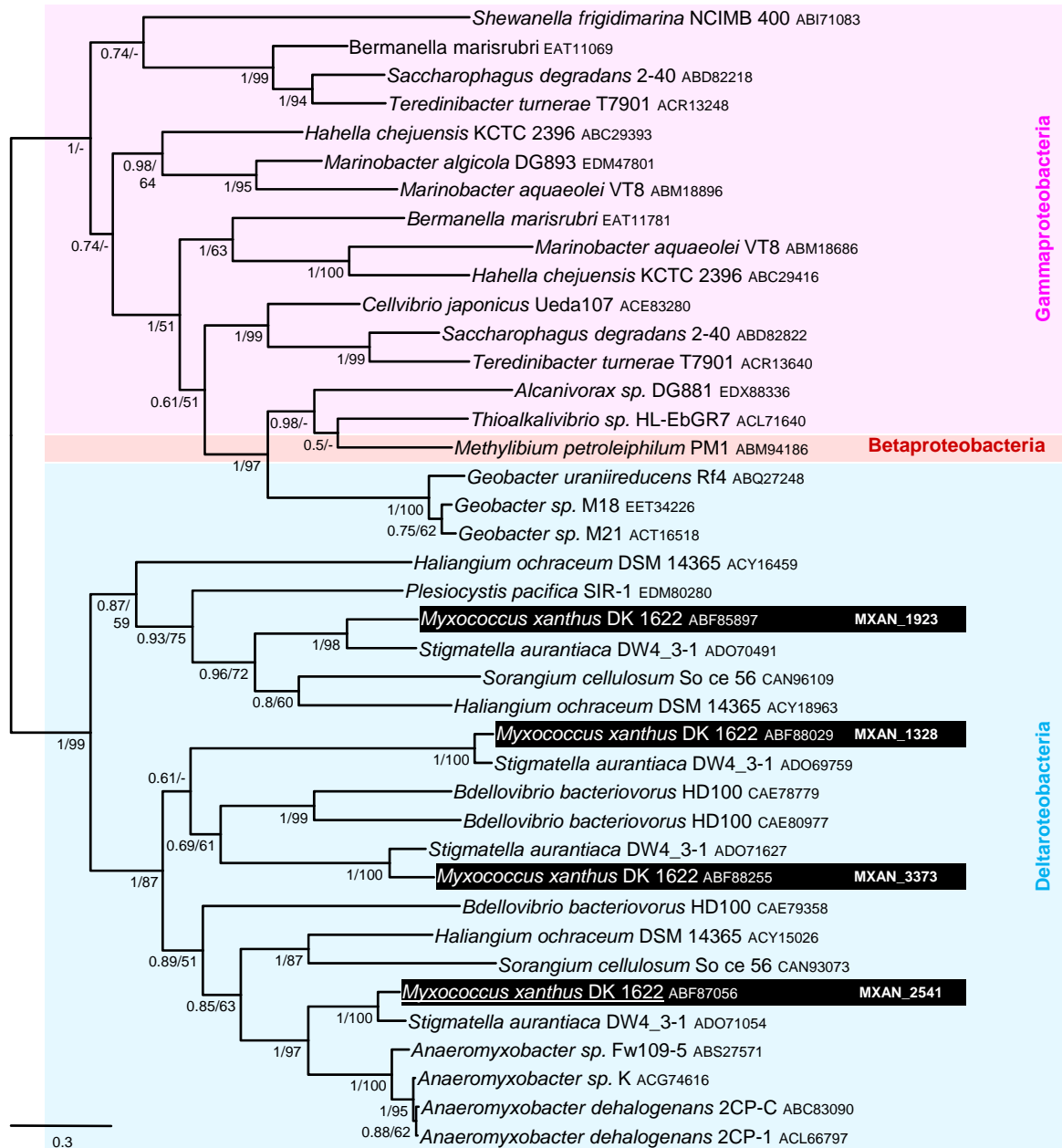

E

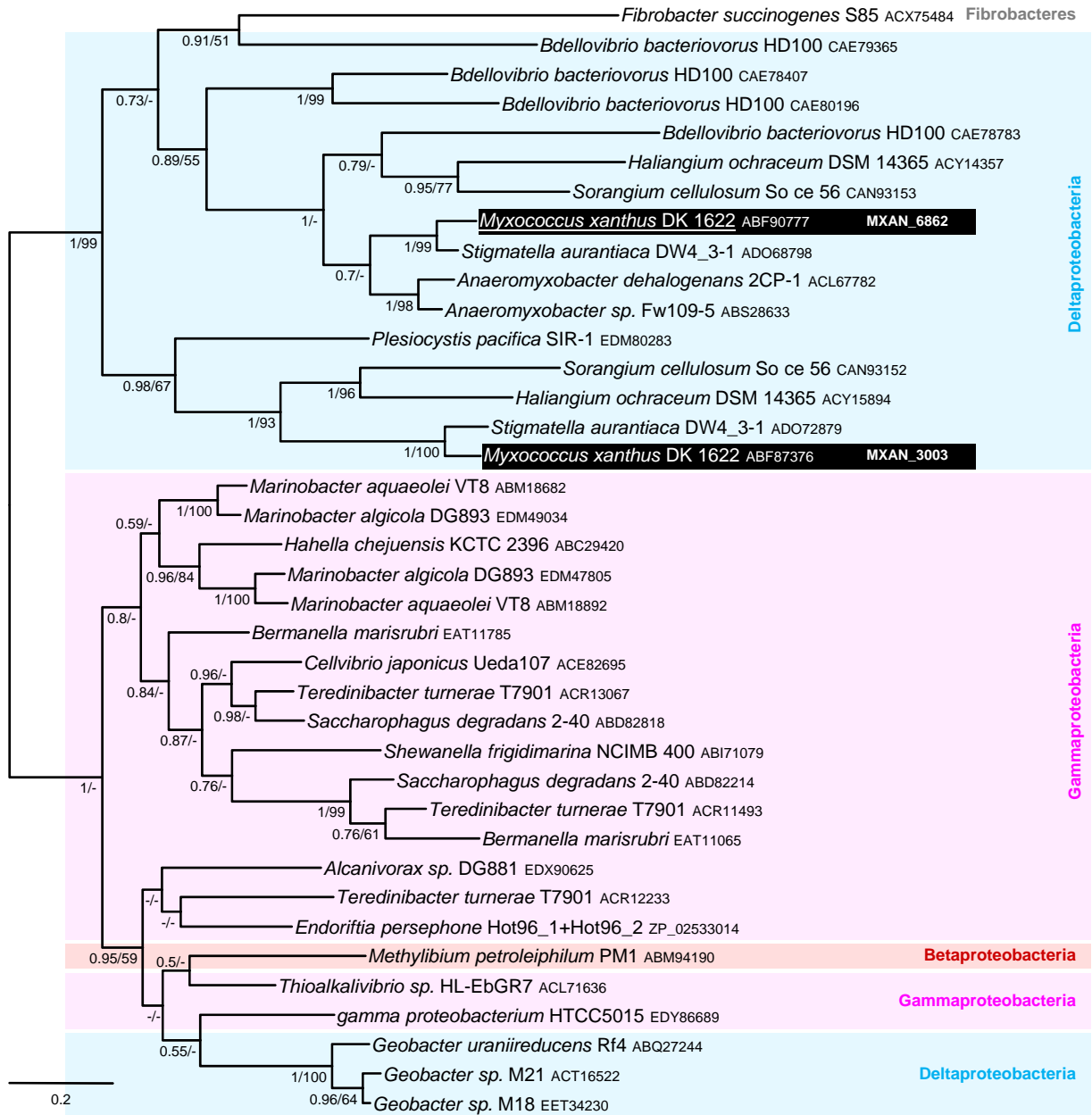

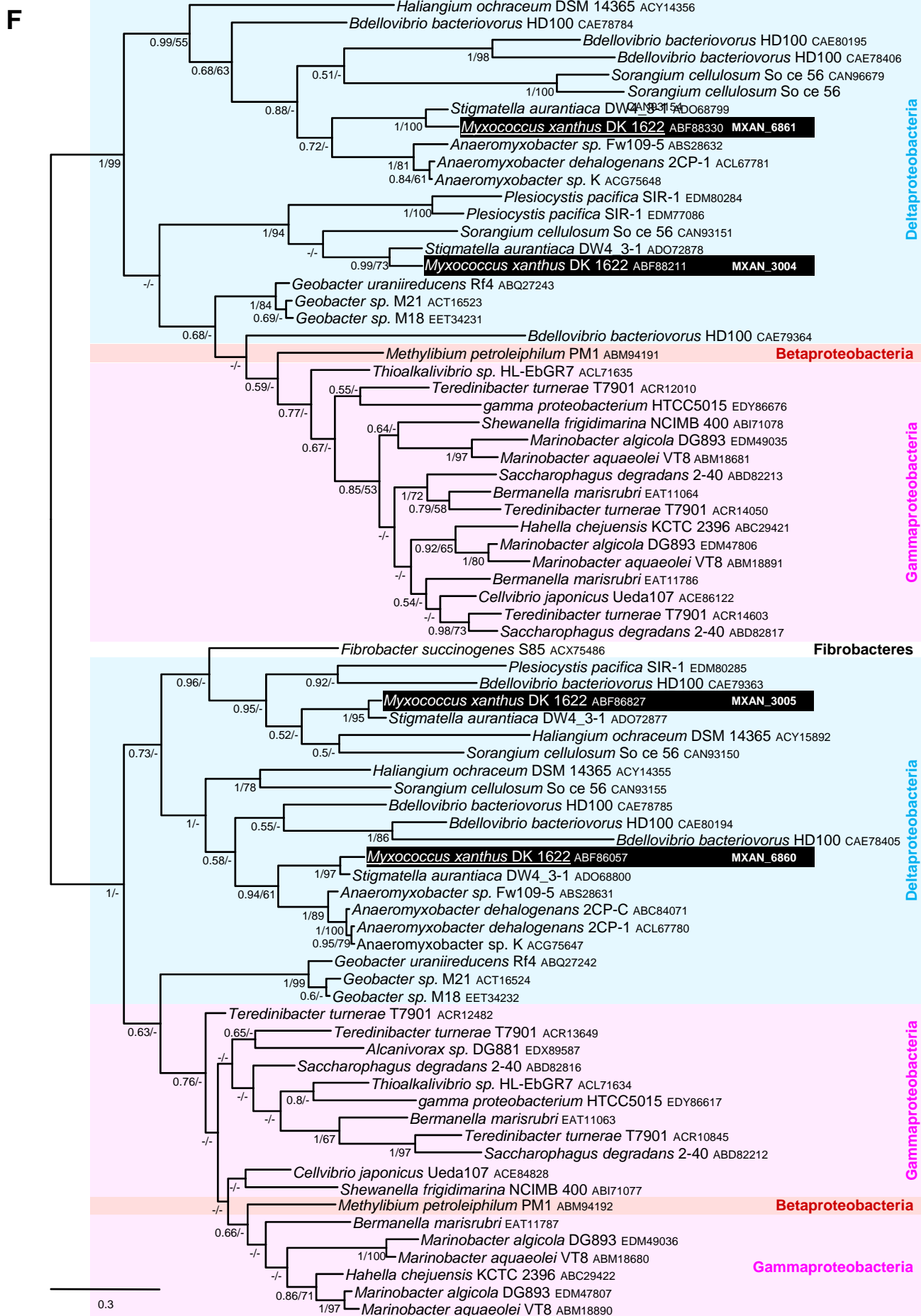

Supplement: Figure S4 — Rooted Bayesian phylogenetic trees (A) of AgmU/GltD (MXAN_4870, 43 sequences, 379 positions), (B) of AglT/GltE (MXAN_4869, 37 sequences, 109 positions), (C) of PglI/GltG (MXAN_4867, 47 sequences, 78 positions), (D) of AgnA/GltC (MXAN_2541, 40 sequences, 185 positions), (E) of AglR (MXAN_6862, 38 sequences, 184 positions) and (F) of AglQ-AglS (MXAN_6861-6860, 76 sequences and 86 positions). The root has been placed accordingly to phylogenies based on whole gene families (not shown). Number at nodes indicates posterior probabilities (PP) and bootstrap support (BS) computed by Mrbayes and PhyMl, respectively. Only posterior probabilities and bootstrap values greater, respectively, than 0.5 and 50 % are shown. The scale bars represent the number of substitutions per site. In each phylogenetic tree proteins putatively involved in gliding in M. xanthus are underlined. (PDF) [file pgen.1002268.s004.pdf]

**WT**

**ΩMXAN1327**

**ΩMXAN1922**

**Ω*nfsD***

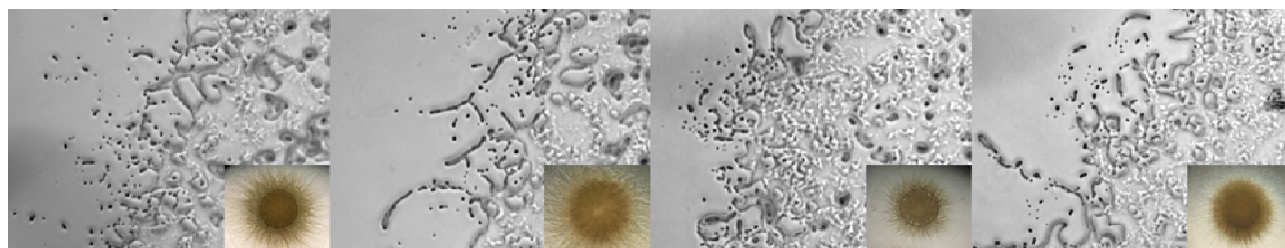

Supplement: Figure S5 — Motility phenotypes of the ΩMxan1327, ΩMxan1922 and ΩnsfD. Colony edges after 48 h incubation on hard (1,5%) agar show WT gliding motility. Insets: twitching motility on soft (0,5%) agar. (PDF) [file pgen.1002268.s005.pdf]
