# Supplementary material for: Emergence and Modular Evolution of a Novel Motility Machinery in Bacteria
Source: PLoS Genet. 2011 Sep 8;7(9):e1002268. doi: 10.1371/journal.pgen.1002268 (PMC3169522; doi:10.1371/journal.pgen.1002268)
Supplement: Table S1 — List of the 23 and 35 Myxococcus xanthus genes identified by two transposon mutagenesis experiments (Yo = [16]; Yu = [17]). For each gene, the locus tag in the genome of M. xanthus, the accession number of the corresponding protein in the ref_seq database and the original functional annotation are provided. For each protein, we indicated the presence of functional conserved domains, of signal peptide and of transmembrane domains. $ signs design false positive genes that have been removed from the current version of the M. xanthus genome. Asterisks correspond to the 28 genes fitting our criteria as putative components of the gliding machinery. Among them we showed that nine (in red) co-localize in three small genomic regions. (PDF) [file pgen.1002268.s006.pdf]

Table S1. List of the 23 and 35 *Myxococcus xanthus* genes identified by two transposon mutagenesis experiments (Yo = [16]; Yu = [17]). For each gene, the locus_tag in the genome of *M. xanthus*, the accession number of the corresponding protein in the ref_seq database and the original functional annotation are provided. For each protein, we indicated the presence of functional conserved domains, of signal peptide and of transmembrane domains. $ signs design false positive genes that have been removed from the current version of the *M. xanthus* genome. Asterisks correspond to the 28 genes fitting our criteria as putative components of the gliding machinery. Among them we showed that nine (in red) co-localize in three small genomic regions.

	**Locus_tag/ Ref_seq**	**Gene**	**Lengh (aa)**	**Former annotation**	**Domain function (pfam)**	**Signal peptide**	**Transmembrane domain**	
**Yo**	($)	agmB	-	ATP-dependent RNA helicase NP643917, *Xanthomonas axonopodis*	-	-	-	
**Yo**	MXAN_0635/ YP_628903	agmE	319	Soj/Par CAB16134, *Bacillus subtilis*	CobQ/CobB/MinD/ParA nucleotide binding domain	no	no	
**Yo**	MXAN_1673/ YP_629925	agmN	513	Hypothetical protein BAB99656, *Corynebacterium glutamicum*	-	no	no	
**Yo**	MXAN_2538/ YP_630757	agmO	170	Proto-oncogene tyrosine-protein kinase c-ABL CAA34438, *Homo sapiens*	-	yes	no	*
**Yo**	MXAN_2542/ YP_630761	agmP	427	Multidrug resistance membrane protein, *Chlorobium tepidum*	Major Facilitator Superfamily	no	10	*
**Yo**	MXAN_2923/ YP_631134	agmQ	698	Leucine aminopeptidase-rekated protein AAF96710, *Vibrio cholerae*	Bacterial pre-peptidase C-terminal domain	no	no	
**Yo**	MXAN_3352/ YP_631549	agmF	346	Pseudouridylate synthase AAM24749, *Thermoanaerobacter tengcongensis*	RNA pseudouridylate synthase	no	no	
**Yo**	MXAN_3502/ YP_631696	agmI	304	Hypothetical protein ZP00079645, *Geobacter metallireducens*	Sporulation related domain	no	1	*
**Yo**	MXAN_3537/ YP_631730	agmL	432	Isocitrate dehydrogenase BAB06878, *Bacillus halodurans*	socitrate/isopropylmalate dehydrogenase	no	no	
**Yo**	MXAN_3842/ YP_632024	agmD	347	tRNA synthetase CAB74224, *Campylobacter jejuni*	tRNA synthetases class I (W and Y)	no	no	
**Yo**	MXAN_3886/ YP_632066	agmA	599	N-acetylmuramoyl L-alanine amidase CAB73523, *Campylobacter jejuni*	N-acetylmuramoyl-L-alanine amidase	yes	no	*
**Yo**	MXAN_4638/ YP_632804	agmH	279	Lysophospholipase ANN50803, *Leptospira interrogens*	Putative lysophospholipase	no	no	
**Yo**	MXAN_4798/ YP_632959	agmC	1731	Putative haemagglutinin/haemolysin-related protein CAD18331, *Ralstonia solanacearum*	Protein of unknown function (DUF3607)	no	1	*
**Yo**	MXAN_4865/ AAO66319 ($)	agmV	1151	No significant similarity	-	no	no	
**Yo**	MXAN_4869/ YP_633027	aglT	478	Predicted N-acetylglucosaminyl tranferase (AGR99) AAH42083, *Homo sapiens*	Tetratricopeptide repeat	yes	no	*
**Yo**	MXAN_4870/ YP_633028	agmU	1218	TPR-domain containing protein AAM05026, *Methanosarcina acetivorans*	Tetratricopeptide repeat	yes	no	*
**Yo**	MXAN_5715/ YP_633853	agmZ	399	No significant similarity	Response regulator receiver domain Histidine kinase-, DNA gyrase B-, and HSP90-like ATPase	no	no	
**Yo**	MXAN_5744/ YP_633881	agmW	458	Carboxy-terminal protease BAC45699, *Bradyrhizobium japonicum*	PDZ domain (Also known as DHR or GLGF) Peptidase family S41	yes	no	*
**Yo**	MXAN_5753/ YP_633890	aglX	236	TolQ biopolymer transport protein I64064, *Haemophilus influenza*	MotA/TolQ/ExbB proton channel family	no	3	*
**Yo**	MXAN_5754/ YP_633891	aglV	153	ExbD/TolR AAM71873, *Chlorobium tepidum*	Biopolymer transport protein ExbD/TolR	no	1	*
**Yo**	MXAN_5756/ YP_633893	aglW	431	TolB AAM71875, *C. tepidum*	TolB amino-terminal domain WD40-like Beta Propeller Repeat	yes	no	*
**Yo**	MXAN_5818/ YP_633955	agmR	371	Putative ion transporting ATPase CAB45559, *Streptomyces coelicolor*	Anion-transporting ATPase	no	no	
**Yo**	MXAN_5820/ YP_633957	agmM	360	Putative metalloprotease C69831, *Bacillus subtilis*	Peptidase family M48	no	5	*
**Yo**	MXAN_6259/ YP_634388	agmJ	636	ABC transporter BAB59028, *Penicillium digitatum*	Protein of unknown function (DUF2629)	no	no	
**Yo**	MXAN_6519/ YP_634642	agmG	712	Site-specific recombinase CAD14714, *Ralstonia solanacearum*	Site-specific recombinase	no	7	*
**Yo**	MXAN_6608/ YP_634726	agmS	263	Enoyl-CoA hydratase AE011275, *Leptospira interrogans*	Enoyl-CoA hydratase/isomerase family	no	no	
**Yo**	MXAN_6860/ YP_634977	aglS	194	ExbD/TolR ACC45288, *Neisseria gonorrhoea*	Biopolymer transport protein ExbD/TolR	no	1	*
**Yo**	MXAN_6862/ YP_634979	aglR	245	MotA/TolQ/ExbB proton channel AAM72811, *Chlorobium tepidum*	MotA/TolQ/ExbB proton channel family protein	no	3	*
**Yo/Yu**	MXAN_6607/ YP_634725	agmT	339	Predicted periplasmic solute-binding protein (*Trichodesmium erythraeum*)	YceG-like family	yes	no	*
**Yo/Yu**	MXAN_1925/ YP_630169	mglA	195	SAR1-like small GTPase / GTPase Sar1p, CAA35978, *Saccharomyces cerevisiae*	ADP-ribosylation factor family	no	no	
**Yo/Yu**	MXAN_1926/ YP_630170	mglB	159	Guanine nucleotide exchange factor for MglA / Ca2+ binding calreticulin AF340232, *Taenia solium*; roadblock-LC7 domain	Roadblock/LC7 domain	no	no	
**Yo/Yu**	MXAN_3008/ YP_631217	aglU	547	WD-repeat lipoprotein, acylaminoacyl-peptidase / b-transducin-like protein HET-E2C AAL37299, Podospora anserina; TolB *M.acetivorans*	WD40-like Beta Propeller Repeat	yes	no	*
**Yo/Yu**	MXAN_3060/ YP_631268	cglB	416	Outer membrane lipoprotein / No significant similarity	-	yes	no	*
**Yo/Yu**	MXAN_4862/ YP_633021	agmX	674	Outer membrane lipoprotein / Putative DnaJ-domain containing protein AC018929, *Oryza sativa*	-	no	1	*
**Yo/Yu**	MXAN_4863/ YP_633022	agmK	3822	TPR repeat protein / TPR protein AAF31047, *Leishmania major*	Tetratricopeptide repeat	no	no	*

	Locus_tag/ Ref_seq	Gene	Lengh (aa)	Former annotation	Domain function (pfam)	Signal peptide	Transmembrane domain	
**Yu**	MXAN_2050/ YP_630279	pglH	927	TPR repeat, CheY-like receiver domain, and a winged-helix DNA-binding domain	Response regulator receiver domain	no	no	
**Yu**	MXAN_2541/ YP_630760	agnA	673	Unknown	Tetratricopeptide repeat	yes	no	*
**Yu**	MXAN_2919/ YP_631130	pglJ	441	Integral membrane protein similar to *S. coelicolor* gi:1098142 with local similarity to Wzy_C polymerase	O-Antigen ligase	no	8	*
**Yu**	MXAN_2921/ YP_631132	pglB	381	Glycosyltranferase similar to YP_000604 of *Leptospira interrogans*	Glycosyl transferases group 1	no	no	
**Yu**	MXAN_4148/ YP_632323	pglK	302	Predicted transmembrane transcriptional regulator	-	no	1	*
**Yu**	MXAN_4616/ YP_632784	pglF	750	Glycosyltranferase 1 domain and glycosyltranferase 2 domain	Starch synthase catalytic domain<br>Glycosyl transferases group 1<br>Glycosyl transferase family 2	no	no	
**Yu**	MXAN_4710/ YP_632872	pglN	328	ADP-heptose synthase, bifunctional sugar kinase/adenylyltranferase RfaE_like	pfkB family carbohydrate kinase	no	no	
**Yu**	MXAN_4867/ YP_633025	pglI	640	Hypothetical abductin-like protein	FHA domain<br>Gram-negative bacterial tonB protein	no	1	*
**Yu**	MXAN_5319/ YP_633470	pglC	841	TPR repeat	Tetratricopeptide repeat	no	no	*
**Yu**	MXAN_5382/ NC_008095	aspT	77pb	tRNA-Asp	tRNA-Asp			
**Yu**	MXAN_5585/ YP_633724	pglE	727	Glycosyltransferase of PMT family	Dolichyl-phosphate-mannose-protein mannosyltransferase	yes	17	*
**Yu**	MXAN_6403/ YP_634527	agnB	408	ABC-type transporter permease protein (*Vibrio fischeri*)	Predicted permease	yes	3	*
**Yu**	MXAN_6501/ YP_634624	pglD	354	GDP-mannose synthesis	Nucleotidyl transferase<br>Mannose-6-phosphate isomerase	no	no	
**Yu**	MXAN_7160/ YP_635273	pglM	409	Alanine racemase	Alanine racemase, N-terminal domain<br>Alanine racemase, C-terminal domain	no	no	
**Yu**	MXAN_7252/ YP_635365	pglA	222	Exopolysaccharide synthesis, ExoD	Exopolysaccharide synthesis, ExoD	no	3	*
**Yu**	MXAN_7296/ YP_635409	agnC	139	Unknown	Protein of unknown function (DUF3478)	no	1	*
