# Supplementary material for: Emergence and Modular Evolution of a Novel Motility Machinery in Bacteria
Source: PLoS Genet. 2011 Sep 8;7(9):e1002268. doi: 10.1371/journal.pgen.1002268 (PMC3169522; doi:10.1371/journal.pgen.1002268)
Supplement: Table S2 — List of complete genomes of Deltaproteobacteria, Gammaproteobacteria, Betaproteobacteria and Fibrobacteres carrying homologues of the 14 candidates genes coding for the gliding machinery in M. xanthus. For each genome, the accession number in the nucleic ref_seq and in the GenBank databases, the size (in megabases) and the release date are provided. (PDF) [file pgen.1002268.s007.pdf]

Table S2. List of complete genomes of Deltaproteobacteria, Gammaproteobacteria, Betaproteobacteria and Fibrobacteres carrying homologues of the 14 candidates genes coding for the gliding machinery in *M. xanthus* . For each genome, the accession number in the nucleic ref_seq and in the GenBank databases, the size (in megabases) and the release date are provided.

Genome	Phylum	Size	GenBank	Ref_Seq	Released
Anaeromyxobacter dehalogenans **2CP-1**	Deltaproteobacteria	5.02	CP001359.1	NC_011891.1	01/12/2009
Anaeromyxobacter dehalogenans **2CP-C**	Deltaproteobacteria	5	CP000251.1	NC_007760.1	01/27/2006
Anaeromyxobacter sp. **Fw 109-5**	Deltaproteobacteria	5.27	CP000769.1	NC_009675.1	07/17/2007
Anaeromyxobacter sp. **K**	Deltaproteobacteria	5.06	CP001131.1	NC_011145.1	08/15/2008
Bdellovibrio bacteriovorus **HD100**	Deltaproteobacteria	3.8	BX842601.2	NC_005363.1	01/31/2004
Geobacter sp. **M21**	Deltaproteobacteria	4.74	CP001661.1	NC_012918.1	06/20/2008
Geobacter uraniireducens **Rf4**	Deltaproteobacteria	5.13	CP000698.1	NC_009483.1	05/11/2007
Haliangium ochraceum **DSM 14365**	Deltaproteobacteria	9.4	CP001804.1	NC_013440.1	04/21/2009
Myxococcus xanthus **DK 1622**	Deltaproteobacteria	9.13	CP000113.1	NC_008095.1	06/07/2006
Sorangium cellulosum **Soce56**	Deltaproteobacteria	13.03	AM746676.1	NC_010162.1	11/27/2007
Stigmatella aurantiaca **DW4/3-1**	Deltaproteobacteria	10	CP002271.1	NC_014623.1	10/21/2010
Fibrobacter succinogenes **S85**	Fibrobacteres	3.8	CP002158	-	08/06/2010
Methylibium petroleiphilum **PM1**	Betaproteobacteria	4.64	CP000555.1	NC_008825.1	01/29/2007
Cellvibrio japonicus **Ueda107**	Gammaproteobacteria	4.57	CP000934.1	NC_010995.1	06/19/2008
Hahella chejuensis KCTC 2396	Gammaproteobacteria	7.2	CP000155.1	NC_007645.1	12/14/2005
Marinobacter aquaeolei **VT8**	Gammaproteobacteria	4.77	CP000514.1	NC_008740.1	12/28/2006
Saccharophagus degradans **2-40**	Gammaproteobacteria	5.05	CP000282.1	NC_007912.1	03/17/2006
Shewanella frigidimarina **NCIMB 400**	Gammaproteobacteria	4.84	CP000447.1	NC_008345.1	09/14/2006
Teredinibacter turnerae **T7901**	Gammaproteobacteria	5.2	CP001614.2	NC_012997.1	05/18/2009
Thioalkalivibrio sp. **HL-EbGR7**	Gammaproteobacteria	3.46	CP001339.1	NC_011901.1	01/13/2009
