# Supplementary material for: Emergence and Modular Evolution of a Novel Motility Machinery in Bacteria
Source: PLoS Genet. 2011 Sep 8;7(9):e1002268. doi: 10.1371/journal.pgen.1002268 (PMC3169522; doi:10.1371/journal.pgen.1002268)
Supplement: Table S3 — Exhaustive list of the homologues of the genes of the G1, G2 and M1 clusters found in complete genomes listed in Table S2. For each gene, the locus_tag, and the accession number, the length and the functional annotation of the corresponding proteins according to the ref_seq database are provided. (PDF) [file pgen.1002268.s008.pdf]

Table S3. Exhaustive list of the homologues of the genes of the G1, G2 and M1 clusters found in complete genomes listed in Table S2. For each gene, the locus_tag, and the accession number, the length and the functional annotation of the corresponding proteins according to the ref_seq database are provided.

Organism	Locus_tag	Ref_seq	Lengh	Annotation	Homolog to
Anaeromyxobacter dehalogenans 2CP-C	Adeh_3310	YP_466514	605	zinc finger/thioredoxin putative	MXAN_4862
	Adeh_3311	YP_466515	4074	TPR repeat-containing protein	MXAN_4863
	Adeh_3318	YP_466522	205	hypothetical protein Adeh_3318	MXAN_4866
	Adeh_3319	YP_466523	673	FHA domain-containing protein	MXAN_4867
	Adeh_3320	YP_466524	89	hypothetical protein Adeh_3320	MXAN_4868
	Adeh_3321	YP_466525	453	TPR repeat-containing protein	MXAN_4869
	Adeh_3322	YP_466526	1193	TPR repeat-containing protein	MXAN_4870
	Adeh_3323	YP_466527	580	hypothetical protein Adeh_3323	MXAN_2541
	Adeh_3324	YP_466528	261	hypothetical protein Adeh_3324	MXAN_2541
	Adeh_3325	YP_466529	291	hypothetical protein Adeh_3325	MXAN_2539
	Adeh_3326	YP_466530	139	hypothetical protein Adeh_3326	MXAN_2538
	Adeh_4308	YP_467508	194	adventurous gliding motility protein S	MXAN_6860
	Adeh_4309	YP_467509	162	TolR protein	MXAN_6861
	Adeh_4310	YP_467510	224	MotA/TolQ/ExbB proton channel	MXAN_6862
	A2cp1_3454	YP_002493850	588	MJ0042 family finger-like protein	MXAN_4862
	A2cp1_3455	YP_002493851	4104	Tetratricopeptide TPR_2 repeat protein	MXAN_4863
	A2cp1_3462	YP_002493858	205	hypothetical protein A2cp1_3462	MXAN_4866
	A2cp1_3463	YP_002493859	680	FHA domain containing protein	MXAN_4867
	A2cp1_3464	YP_002493860	89	hypothetical protein A2cp1_3464	MXAN_4868
	A2cp1_3465	YP_002493861	451	Tetratricopeptide TPR_2 repeat protein	MXAN_4869

Anaeromyxobacter dehalogenans 2CP-1	A2cp1_3466	YP_002493862	1191	TPR repeat-containing protein	MXAN_4870
	A2cp1_3467	YP_002493863	580	hypothetical protein A2cp1_3467	MXAN_2541
	A2cp1_3468	YP_002493864	261	hypothetical protein A2cp1_3468	MXAN_2541
	A2cp1_3469	YP_002493865	284	hypothetical protein A2cp1_3469	MXAN_2539
	A2cp1_3470	YP_002493866	139	hypothetical protein A2cp1_3470	MXAN_2538
	A2cp1_4463	YP_002494846	194	Biopolymer transport protein ExbD/TolR	MXAN_6860
	A2cp1_4464	YP_002494847	162	Biopolymer transport protein ExbD/TolR	MXAN_6861
	A2cp1_4465	YP_002494848	224	MotA/TolQ/ExbB proton channel	MXAN_6862
Anaeromyxobacter sp. FW 109-5	Anae109_3371	YP_001380539	571	Zinc finger-domain-containing protein	MXAN_4862
	Anae109_3372	YP_001380540	4095	TPR repeat-containing protein	MXAN_4863
	Anae109_3382	YP_001380550	205	hypothetical protein Anae109_3382	MXAN_4866
	Anae109_3383	YP_001380551	667	FHA domain-containing protein	MXAN_4867
	Anae109_3384	YP_001380552	90	hypothetical protein Anae109_3384	MXAN_4868
	Anae109_3385	YP_001380553	427	TPR repeat-containing protein	MXAN_4869
	Anae109_3386	YP_001380554	1162	TPR repeat-containing protein	MXAN_4870
	Anae109_3387	YP_001380555	649	hypothetical protein Anae109_3387	MXAN_2541
	Anae109_3388	YP_001380556	254	hypothetical protein Anae109_3388	MXAN_2541
	Anae109_3389	YP_001380557	285	hypothetical protein Anae109_3389	MXAN_2539
	Anae109_3390	YP_001380558	172	hypothetical protein Anae109_3390	MXAN_2538
	Anae109_4453	YP_001381615	194	adventurous gliding motility protein S	MXAN_6860
	Anae109_4454	YP_001381616	164	TolR protein	MXAN_6861
	Anae109_4455	YP_001381617	226	MotA/TolQ/ExbB proton channel	MXAN_6862
	AnaeK_3390	YP_002135733	610	MJ0042 family finger-like protein	MXAN_4862
	AnaeK_3398	YP_002135740	205	hypothetical protein AnaeK_3398	MXAN_4866
	AnaeK_3399	YP_002135741	682	FHA domain containing protein	MXAN_4867

Anaeromyxobacter sp. K	AnaeK_3400	YP_002135742	89	hypothetical protein AnaeK_3400	MXAN_4868
	AnaeK_3401	YP_002135743	453	Tetratricopeptide TPR_4	MXAN_4869
	AnaeK_3402	YP_002135744	1192	Tetratricopeptide TPR_2 repeat protein	MXAN_4870
	AnaeK_3403	YP_002135745	580	hypothetical protein AnaeK_3403	MXAN_2541
	AnaeK_3404	YP_002135746	261	hypothetical protein AnaeK_3404	MXAN_2541
	AnaeK_3405	YP_002135747	284	hypothetical protein AnaeK_3405	MXAN_2539
	AnaeK_3406	YP_002135748	139	hypothetical protein AnaeK_3406	MXAN_2538
	AnaeK_4444	YP_002136776	194	adventurous gliding motility protein S	MXAN_6860
	AnaeK_4445	YP_002136777	162	TolR protein	MXAN_6861
	AnaeK_4446	YP_002136778	224	MotA/TolQ/ExbB proton channel	MXAN_6862
	Bd0413	NP_967407	355	hypothetical protein Bd0413	MXAN_2541
	Bd0414	NP_967408	246	hypothetical protein Bd0414	MXAN_2539
	Bd0416	NP_967410	408	hypothetical protein Bd0416	MXAN_4867
	Bd0417	NP_967411	987	hypothetical protein Bd0417	MXAN_4870
	Bd0418	NP_967412	165	hypothetical protein Bd0418	MXAN_6860
	Bd0419	NP_967413	149	putative adventurous gliding motility protein V	MXAN_6861
	Bd0420	NP_967414	248	putative adventurous gliding motility protein R	MXAN_6862
	Bd0828	NP_967784	240	hypothetical protein Bd0828	MXAN_2541
	Bd0829	NP_967785	266	hypothetical protein Bd0829	MXAN_2539
	Bd0831	NP_967786	529	hypothetical protein Bd0831	MXAN_2541
	Bd0832	NP_967787	1066	adventurous gliding motility protein U	MXAN_4870
	Bd0833	NP_967788	281	adventurous gliding motility protein T	MXAN_4869
	Bd0834	NP_967789	707	hypothetical protein Bd0834	MXAN_4867
	Bd0836	NP_967790	221	adventurous gliding motility protein R	MXAN_6862
	Bd0837	NP_967791	158	tolR protein	MXAN_6861

Bdellovibrio bacteriovorus HD100	Bd0838	NP_967792	196	adventurous gliding motility protein S	MXAN_6860
	Bd1474	NP_968365	713	hypothetical protein Bd1474	MXAN_2541
	Bd1475	NP_968366	254	hypothetical protein Bd1475	MXAN_2539
	Bd1476	NP_968367	488	hypothetical protein Bd1476	MXAN_4867
	Bd1477	NP_968368	82	hypothetical protein Bd1477	MXAN_4868
	Bd1478	NP_968369	1237	adventurous gliding motility protein U	MXAN_4870
	Bd1479	NP_968370	167	adventurous gliding motility protein S	MXAN_6860
	Bd1480	NP_968371	185	TolR-like protein, putative	MXAN_6861
	Bd1481	NP_968372	236	adventurous gliding motility protein R	MXAN_6862
	Bd2369	NP_969195	531	hypothetical protein Bd2369	MXAN_2541
	Bd2370	NP_969196	235	hypothetical protein Bd2370	MXAN_2539
	Bd2372	NP_969198	376	hypothetical protein Bd2372	MXAN_4867
	Bd2374	NP_969200	996	hypothetical protein Bd2374	MXAN_4870
	Bd2375	NP_969201	175	adventurous gliding motility protein S	MXAN_6860
	Bd2376	NP_969202	151	hypothetical protein Bd2376	MXAN_6861
	Bd2377	NP_969203	244	adventurous gliding motility protein R	MXAN_6862
	Bd3598	NP_970322	278	hypothetical protein Bd3598	MXAN_2539
	Bd3599	NP_970323	513	hypothetical protein Bd3599	MXAN_2541
	Bd3600	NP_970324	52	hypothetical protein Bd3600	MXAN_4868
	Bd3601	NP_970325	239	hypothetical protein Bd3601	MXAN_2541
Geobacter uraniireducens Rf4	Gura_3070	YP_001231814	484	hypothetical protein Gura_3070	MXAN_4867
	Gura_3071	YP_001231815	166	hypothetical protein Gura_3071	MXAN_6860
	Gura_3072	YP_001231816	166	biopolymer transport protein ExbD/TolR	MXAN_6861
	Gura_3073	YP_001231817	211	MotA/TolQ/ExbB proton channel	MXAN_6862
	Gura_3075	YP_001231819	240	TPR repeat-containing protein	MXAN_4869

	Gura_3076	YP_001231820	1108	TPR repeat-containing protein	MXAN_4870
	Gura_3077	YP_001231821	629	hypothetical protein Gura_3077	MXAN_2541
Geobacter sp. M21	GM21_0438	YP_003020276	626	hypothetical protein GM21_0438	MXAN_2541
	GM21_0439	YP_003020277	1090	Tetratricopeptide domain protein	MXAN_4870
	GM21_0440	YP_003020278	272	TPR repeat-containing protein	MXAN_4869
	GM21_0442	YP_003020280	211	MotA/TolQ/ExbB proton channel	MXAN_6862
	GM21_0443	YP_003020281	166	Biopolymer transport protein ExbD/TolR	MXAN_6861
	GM21_0444	YP_003020282	165	Biopolymer transport protein ExbD/TolR	MXAN_6860
	GM21_0445	YP_003020283	505	hypothetical protein GM21_0445	MXAN_4867
Haliangium ochraceum DSM 14365	Hoch_1807	YP_003266248	201	Biopolymer transport protein ExbD/TolR	MXAN_6860
	Hoch_1808	YP_003266249	163	Biopolymer transport protein ExbD/TolR	MXAN_6861
	Hoch_1809	YP_003266250	216	MotA/TolQ/ExbB proton channel	MXAN_6862
	Hoch_2490	YP_003266919	560	Tetratricopeptide repeat protein	MXAN_2541
	Hoch_2491	YP_003266920	1155	Tetratricopeptide repeat protein	MXAN_4870
	Hoch_2492	YP_003266921	441	Tetratricopeptide TPR_2 repeat protein	MXAN_4869
	Hoch_3390	YP_003267785	187	Biopolymer transport protein ExbD/TolR	MXAN_6860
	Hoch_3391	YP_003267786	242	Biopolymer transport protein ExbD/TolR	MXAN_6861
	Hoch_3392	YP_003267787	223	MotA/TolQ/ExbB proton channel	MXAN_6862
	Hoch_3957	YP_003268349	228	hypothetical protein Hoch_3957	MXAN_2539
	Hoch_3958	YP_003268350	268	hypothetical protein Hoch_3958	MXAN_2541
	Hoch_3960	YP_003268352	689	hypothetical protein Hoch_3960	MXAN_2541
	Hoch_3961	YP_003268353	1058	Tetratricopeptide TPR_2 repeat protein	MXAN_4870
	Hoch_3962	YP_003268354	395	Tetratricopeptide TPR_2 repeat protein	MXAN_4869
	Hoch_3963	YP_003268355	185	hypothetical protein Hoch_3963	MXAN_4868
	Hoch_3964	YP_003268356	474	TonB family protein	MXAN_4867

	Hoch_5625	YP_003269998	296	Tetratricopeptide TPR_2 repeat protein	MXAN_4869
	Hoch_5627	YP_003269999	767	FHA domain containing protein	MXAN_4867
	Hoch_5628	YP_003270000	84	hypothetical protein Hoch_5628	MXAN_4868
	Hoch_6494	YP_003270856	823	hypothetical protein Hoch_6494	MXAN_2541
	Hoch_6495	YP_003270857	1202	Tetratricopeptide repeat protein	MXAN_4870
	Hoch_6496	YP_003270858	422	hypothetical protein Hoch_6496	MXAN_4869
	Hoch_6497	YP_003270859	120	hypothetical protein Hoch_6497	MXAN_4868
	Hoch_6498	YP_003270860	490	hypothetical protein Hoch_6498	MXAN_4867
Myxococcus xanthus DK622	MXAN_1327	YP_629584	1089	TPR repeat-containing protein	MXAN_4870
	MXAN_1328	YP_629585	538	hypothetical protein MXAN_1328	MXAN_2541
	MXAN_1329	YP_629586	246	hypothetical protein MXAN_1329	MXAN_2539
	MXAN_1330	YP_629587	749	FHA domain-containing protein	MXAN_4867
	MXAN_1331	YP_629588	162	hypothetical protein MXAN_1331	MXAN_4868
	MXAN_1919	YP_630163	447	hypothetical protein MXAN_1919	MXAN_2541
	MXAN_1920	YP_630164	91	hypothetical protein MXAN_1920	MXAN_4870
	MXAN_1921	YP_630165	490	TPR repeat-containing protein	MXAN_4869
	MXAN_1922	YP_630166	1111	TPR repeat-containing protein	MXAN_4868
	MXAN_1923	YP_630167	809	hypothetical protein MXAN_1923	MXAN_4867
	MXAN_3003	YP_631212	260	MotA/TolQ/ExbB proton channel family protein	MXAN_6862
	MXAN_3004	YP_631213	173	hypothetical protein MXAN_3004	MXAN_6861
	MXAN_3005	YP_631214	193	hypothetical protein MXAN_3005	MXAN_6860
	MXAN_3371	YP_631568	294	hypothetical protein MXAN_3371	MXAN_2541
	MXAN_3372	YP_631569	442	hypothetical protein MXAN_3372	MXAN_2539
	MXAN_3373	YP_631570.1	512	hypothetical protein MXAN_3373	MXAN_2541
	MXAN_3374	YP_631571	1219	TPR repeat-containing protein	MXAN_4870

	MXAN_3375	YP_631572	499	putative adventurous gliding protein T	MXAN_4869
	MXAN_3376	YP_631573	96	hypothetical protein MXAN_3376	MXAN_4868
	MXAN_3377	YP_631574	682	FHA/TonB domain-containing protein	MXAN_4867
	MXAN_3378	YP_631575	200	hypothetical protein MXAN_3378	MXAN_4866
Sorangium cellulosum Soce56	sce2906	YP_001613545	292	hypothetical protein sce2906	MXAN_2541
	sce2907	YP_001613546	308	hypothetical protein sce2907	MXAN_2539
	sce2914	YP_001613553	568	hypothetical protein sce2914	MXAN_2541
	sce2915	YP_001613554	1289	hypothetical protein sce2915	MXAN_4870
	sce2916	YP_001613555	497	hypothetical protein sce2916	MXAN_4869
	sce2917	YP_001613556	90	hypothetical protein sce2917	MXAN_4868
	sce2918	YP_001613557	681	FHA domain-containing protein	MXAN_4867
	sce2920	YP_001613559	1774	TPR repeat-containing protein	MXAN_4863
	sce2921	YP_001613560	2081	TPR repeat-containing protein	MXAN_4863
	sce2991	YP_001613630	202	putative adventurous gliding motility protein	MXAN_6860
	sce2992	YP_001613631	187	TolR-like protein	MXAN_6861
	sce2993	YP_001613632	225	adventurous gliding motility protein R	MXAN_6862
	sce2994	YP_001613633	220	adventurous gliding motility protein R	MXAN_6862
	sce2995	YP_001613634	189	putative biopolymer transport protein	MXAN_6861
	sce2996	YP_001613635	169	putative adventurous gliding motility protein AglS	MXAN_6860
	sce5940	YP_001616584	511	hypothetical protein sce5940	MXAN_4867
	sce5942	YP_001616586	82	hypothetical protein sce5942	MXAN_4868
	sce5943	YP_001616587	449	TPR domain-containing protein	MXAN_4869
	sce5944	YP_001616588	1431	hypothetical protein sce5944	MXAN_4870
	sce5945	YP_001616589	802	hypothetical protein sce5945	MXAN_2541
	STAUR_0994	YP_003950625	246	mota/tolq/exbb proton channel family protein	MXAN_6862

Stigmatella aurantiaca DW4/3-1	STAUR_0995	YP_003950626	160	biopolymer transport protein, exbd/tolr family	MXAN_6861
	STAUR_0996	YP_003950627	195	adventurous gliding motility protein agls	MXAN_6860
	STAUR_1954	YP_003951585	1091	tetratricopeptide repeat protein	MXAN_4870
	STAUR_1955	YP_003951586	520	hypothetical protein STAUR_1955	MXAN_2541
	STAUR_1956	YP_003951587	211	hypothetical protein STAUR_1956	MXAN_2539
	STAUR_1957	YP_003951588	745	fha domain protein	MXAN_4867
	STAUR_1958	YP_003951589	133	hypothetical protein STAUR_1958	MXAN_4868
	STAUR_2684	YP_003952314	450	hypothetical protein STAUR_2684	MXAN_4867
	STAUR_2685	YP_003952315	82	hypothetical protein STAUR_2685	MXAN_4868
	STAUR_2686	YP_003952316	442	tetratricopeptide repeat protein	MXAN_4869
	STAUR_2687	YP_003952317	1109	tetratricopeptide repeat protein	MXAN_4870
	STAUR_2688	YP_003952318	785	hypothetical protein STAUR_2688	MXAN_2541
	STAUR_3259	YP_003952878	176	adventurous gliding motility protein agmo	MXAN_2538
	STAUR_3260	YP_003952879	274	hypothetical protein STAUR_3260	MXAN_2539
	STAUR_3261	YP_003952880	270	hypothetical protein STAUR_3261	MXAN_2541
	STAUR_3262	YP_003952881	674	tetratricopeptide repeat domain protein	MXAN_2541
	STAUR_3837	YP_003953452	273	hypothetical protein STAUR_3837	MXAN_2541
	STAUR_3838	YP_003953453	397	hypothetical protein STAUR_3838	MXAN_2539
	STAUR_3839	YP_003953454	511	hypothetical protein STAUR_3839	MXAN_2541
	STAUR_3840	YP_003953455	1209	tetratricopeptide repeat protein	MXAN_4870
	STAUR_3841	YP_003953456	479	tetratricopeptide repeat family protein	MXAN_4869
	STAUR_3842	YP_003953457	97	hypothetical protein STAUR_3842	MXAN_4868
	STAUR_3843	YP_003953458	641	fha/tonb domain protein	MXAN_4867
	STAUR_3845	YP_003953460	198	hypothetical protein STAUR_3845	MXAN_4866
	STAUR_5105	YP_003954704	175	hypothetical protein STAUR_5105	MXAN_6860

	STAUR_5106	YP_003954705	172	hypothetical protein STAUR_5106	MXAN_6861
	STAUR_5107	YP_003954706	244	mota/tolq/exbb proton channel family protein	MXAN_6862
	STAUR_5649	YP_003955239	653	adventurous gliding motility protein agmx	MXAN_4862
	STAUR_5650	YP_003955240	4089	adventurous gliding motility protein agmk	MXAN_4863
	STAUR_5652	YP_003955242	211	hypothetical protein STAUR_5652	MXAN_4866
	STAUR_5653	YP_003955243	640	fha domain/tonb domain protein	MXAN_4867
	STAUR_5654	YP_003955244	88	hypothetical protein STAUR_5654	MXAN_4868
	STAUR_5655	YP_003955245	474	adventurous gliding protein t	MXAN_4869
	STAUR_5656	YP_003955246	1216	tetratricopeptide repeat protein	MXAN_4870
	Fiscuc_1892	YP_003249964	746	TPR repeat-containing protein	MXAN_2541
	Fiscuc_1893	YP_003249965	1292	TPR repeat-containing protein	MXAN_4870
Fibrobacter succinogenes S85	Fiscuc_1894	YP_003249966	212	MotA/TolQ/ExbB proton channel	MXAN_6862
	Fiscuc_1896	YP_003249968	164	Biopolymer transport protein ExbD/TolR	MXAN_6860
	Fiscuc_1897	YP_003249969	308	TonB family protein	MXAN_4867
	Mpe_A1224	YP_001020421	634	hypothetical protein Mpe_A1224	MXAN_2541
	Mpe_A1225	YP_001020422	941	hypothetical protein Mpe_A1225	MXAN_4870
	Mpe_A1226	YP_001020423	243	hypothetical protein Mpe_A1226	MXAN_4869
Methylibium petroleiphilum PM1	Mpe_A1228	YP_001020425	217	putative adventurous gliding motility protein R	MXAN_6862
	Mpe_A1229	YP_001020426	170	hypothetical protein Mpe_A1229	MXAN_6861
	Mpe_A1230	YP_001020427	163	adventurous gliding motility protein S	MXAN_6860
	Mpe_A1231	YP_001020428	371	hypothetical protein Mpe_A1231	MXAN_4867
	CJA_3312	YP_001983766	323	TonB family C-terminal domain protein	MXAN_4867
	CJA_3313	YP_001983767	173	putative adventurous gliding motility protein S	MXAN_6860
	CJA_3314	YP_001983768	181	hypothetical protein CJA_3314	MXAN_6861
Cellvibrio japonicus Ueda107	CJA_3315	YP_001983769	231	adventurous gliding motility protein R	MXAN_6862

	CJA_3317	YP_001983771	244	putative TPR domain protein	MXAN_4869
	CJA_3318	YP_001983772	964	tetratricopeptide repeat domain protein	MXAN_4870
	CJA_3319	YP_001983773	660	hypothetical protein CJA_3319	MXAN_2541
Hahella chejuensis KCTC 2396	HCH_02601	YP_433818	640	TPR repeat-containing protein	MXAN_4869
	HCH_02602	YP_433819	963	hypothetical protein HCH_02602	MXAN_4870
	HCH_02603	YP_433820	203	TPR repeat-containing protein	MXAN_4869
	HCH_02626	YP_433841	584	TPR repeat-containing protein	MXAN_2541
	HCH_02627	YP_433842	933	hypothetical protein HCH_02627	MXAN_4870
	HCH_02628	YP_433843	197	TPR repeat-containing protein	MXAN_4869
	HCH_02631	YP_433845	221	biopolymer transport protein	MXAN_6862
	HCH_02632	YP_433846	170	hypothetical protein HCH_02632	MXAN_6861
	HCH_02633	YP_433847	165	biopolymer transport protein	MXAN_6860
	HCH_02634	YP_433848	334	hypothetical protein HCH_02634	MXAN_4867
Marinobacter aquaeolei VT8	Maqu_1595	YP_958866	321	hypothetical protein Maqu_1595	MXAN_4867
	Maqu_1596	YP_958867	162	biopolymer transport protein	MXAN_6860
	Maqu_1597	YP_958868	176	biopolymer transport protein ExbD/TolR	MXAN_6861
	Maqu_1598	YP_958869	221	MotA/TolQ/ExbB proton channel	MXAN_6862
	Maqu_1600	YP_958871	162	TPR repeat-containing protein	MXAN_4869
	Maqu_1601	YP_958872	939	TPR repeat-containing protein	MXAN_4870
	Maqu_1602	YP_958873	550	hypothetical protein Maqu_1602	MXAN_2541
	Maqu_1807	YP_959076	324	hypothetical protein Maqu_1807	MXAN_4867
	Maqu_1808	YP_959077	166	biopolymer transport protein ExbD/TolR	MXAN_6860
	Maqu_1809	YP_959078	170	biopolymer transport protein ExbD/TolR	MXAN_6861
	Maqu_1810	YP_959079	235	MotA/TolQ/ExbB proton channel	MXAN_6862
	Maqu_1812	YP_959081	189	TPR repeat-containing protein	MXAN_4869

	Maqu_1813	YP_959082	952	TPR repeat-containing protein	MXAN_4870
	Maqu_1814	YP_959083	648	TPR repeat-containing protein	MXAN_2541
Saccharophagus degradans 2-40	Sde_2954	YP_528423	325	hypothetical protein Sde_2954	MXAN_4867
	Sde_2955	YP_528424	185	hypothetical protein Sde_2955	MXAN_6860
	Sde_2956	YP_528425	169	hypothetical protein Sde_2956	MXAN_6861
	Sde_2957	YP_528426	242	biopolymer transport proteins-like	MXAN_6862
	Sde_2959	YP_528428	230	cellulose binding, type IV	MXAN_4869
	Sde_2960	YP_528429	952	cellulose binding, type IV	MXAN_4870
	Sde_2961	YP_528430	570	hypothetical protein Sde_2961	MXAN_2541
	Sde_3560	YP_529027	325	hypothetical protein Sde_3560	MXAN_4867
	Sde_3561	YP_529028	178	sigma-70 factor	MXAN_6860
	Sde_3562	YP_529029	183	hypothetical protein Sde_3562	MXAN_6861
	Sde_3563	YP_529030	220	ATPase	MXAN_6862
	Sde_3565	YP_529032	222	hypothetical protein Sde_3565	MXAN_4869
	Sde_3566	YP_529033	957	coenzyme A biosynthesis protein	MXAN_4870
	Sde_3567	YP_529034	624	hypothetical protein Sde_3567	MXAN_2541
Shewanella frigidimarina NCIMB 400	Sfri_1223	YP_749914	332	hypothetical protein Sfri_1223	MXAN_4867
	Sfri_1224	YP_749915	177	sigma-70 factor	MXAN_6860
	Sfri_1225	YP_749916	188	hypothetical protein Sfri_1225	MXAN_6861
	Sfri_1226	YP_749917	217	MotA/TolQ/ExbB proton channel	MXAN_6862
	Sfri_1228	YP_749919	263	TPR repeat-containing protein	MXAN_4869
	Sfri_1229	YP_749920	1016	TPR repeat-containing protein	MXAN_4870
	Sfri_1230	YP_749921	692	hypothetical protein Sfri_1230	MXAN_2541
	TERTU_1670	YP_003073195	205	MotA/TolQ/ExbB proton channel	MXAN_6862
	TERTU_1671	YP_003073196	173	transport energizing protein, ExbD/TolR family	MXAN_6861

Teredinibacter turnerae T7901	TERTU_1672	YP_003073197	165	hypothetical protein TERTU_1672	MXAN_6860
	TERTU_1849	YP_003073348	552	hypothetical protein TERTU_1849	MXAN_2541
	TERTU_1850	YP_003073349	947	TPR repeat domain protein	MXAN_4870
	TERTU_1851	YP_003073350	212	tetratricopeptide repeat domain protein	MXAN_4869
	TERTU_1853	YP_003073352	272	transporter, MotA/TolQ/ExbB proton channel family	MXAN_6862
	TERTU_1854	YP_003073353	170	hypothetical protein TERTU_1854	MXAN_6861
	TERTU_1855	YP_003073354	178	hypothetical protein TERTU_1855	MXAN_6860
	TERTU_1856	YP_003073355	321	hypothetical protein TERTU_1856	MXAN_4867
	TERTU_3740	YP_003075049	325	TonB domain protein	MXAN_4867
	TERTU_3741	YP_003075050	176	hypothetical protein TERTU_3741	MXAN_6860
	TERTU_3742	YP_003075051	183	hypothetical protein TERTU_3742	MXAN_6861
	TERTU_3743	YP_003075052	245	ATPase	MXAN_6862
	TERTU_3745	YP_003075054	225	tetratricopeptide repeat protein	MXAN_4869
	TERTU_3746	YP_003075055	964	TPR repeat domain protein	MXAN_4870
	TERTU_3747	YP_003075056	640	tetratricopeptide repeat domain protein	MXAN_2541
Thioalkalivibrio sp. HL-EbGR7	Tgr7_0536	YP_002512620	535	hypothetical protein Tgr7_0536	MXAN_4867
	Tgr7_0537	YP_002512621	170	sigma-70 factor	MXAN_6860
	Tgr7_0538	YP_002512622	168	hypothetical protein Tgr7_0538	MXAN_6861
	Tgr7_0539	YP_002512623	222	MotA/TolQ/ExbB proton channel	MXAN_6862
	Tgr7_0541	YP_002512625	190	TPR repeat-containing protein	MXAN_4869
	Tgr7_0542	YP_002512626	922	hypothetical protein Tgr7_0542	MXAN_4870
	Tgr7_0543	YP_002512627	620	hypothetical protein Tgr7_0543	MXAN_2541
