# Supplementary material for: Emergence and Modular Evolution of a Novel Motility Machinery in Bacteria
Source: PLoS Genet. 2011 Sep 8;7(9):e1002268. doi: 10.1371/journal.pgen.1002268 (PMC3169522; doi:10.1371/journal.pgen.1002268)
Supplement: Table S4 — Primers. (PDF) [file pgen.1002268.s009.pdf]

Table S4. Primers

Name	Sequences of primers (5'---3')
1922-1	AAAGAATTCCTGGTACGAGAAGAACAAGGGC
1922-2	AAAAAGCTTGTAGATGCGCTCGTACAGCTTC
3374-1	AAAGAATTCCAGTTGGTCCGCAAGGAAATG
3374-2	AAAAAGCTTCTTCTTCGGGTTCTTCTCCTTC
INS1327-1	CCCAAGCTTTGGAGAAGAGCCGCTACG
INS1327-2	CGGGATCCCGTCGTAGAGCTTCTGG
DAGMU7	AAAGAATTCACGCCTCGCCATTCGC
DAGMU8	AAAGGTACCCATGGTCTTCCTAGGAGAGGGGC
DAGMU9	AAAGGTACCAAGACTCAGGTGGACGCC
DAGMU10	AAAAAGCTTCTGGCCCATGCCCTTGTA
DAGLT1	AAGAATTCGAACGAGTACCGGCGCC
DAGLT5	AAAGGATCCTGGCCTTGGAGGACGTAC
DAGLT6	AAAGGATCCCCGACCAGAAGAATGCAGG
DAGLT4	TTAAGCTTCCGTTGACGTACGTGCCC
D4868-1	AAGAATTCCCGGAGCTCCACCACG
D4868-5	AAAGGATCCTCACAGCAGGTCGTCTTCC
D4868-6	AAAGGATCCTAGCCAGCGCCACAACGC
D4868-4	TTAAGCTTCGAAGGCCCCCTTCCTAC
D4867-1	AAGAATTCCGAAGCGCGAGGCCAAG
D4867-5	AAAGGATCCGTCCTTGGAGGCGACCAG
D4867-6	AAAGGATCCCGTGGTGGTCGTCACGTATC
D4867-4	TTAAGCTTACGTCTTTCTCCCGCTAGAAC
D4866-1	AAGAATTCCACCCGAACAAGAAGGACGAG
D4866-2	GCGGTCCTGACGTTGGC
D4866-3	CAACGTCAGGACCGCCGGGAGAAAGACGTGCCTC
D4866-4	TTAAGCTTTCCTCGGCCTCGGGAC
4866-4	AAATCTAGACGCCGGGTGGCCTTTC
4866-5	AAAAAGCTTGAAATCCGTCTTGGAGAGCGC
D4866-4	TTAAGCTTTCCTCGGCCTCGGGAC
D2541-1	GGAATTCCCTCGGCGCTGCCGGGGCGAG
D2541-2	CGGGATCCGGCGACGCGGATGAGCCGGA
D2541-3	CGGGATCCGGACCCGGGACTTCTCCCCC
D2541-4	CCCAAGCTTGCGCCTCGCGCCCTGGCGCG
PROM4868-1	CCCAAGCTTCGACGCCGGCTTCCGGCACGG
4868-SANSSTOP-2	CAGCTCGCCCACGGACTGCATCACCTTG
4868-MCHERRY-3	GTCCGTGGGCGAGCTGGTGAGCAAGGGCGAGGAG
MCHERRY-4	CGGGATCCTTACTTGTACAGCTCGTCCATGCCG
PROM4867-BAMHI	CGCGGATCCATGCAGTCCGTGGG
4867-ECORI-4	CGGAATTCCCGGCGCTATTCGCCG
2539-1	CGGAATTCAACCAGTCGCGCGGAAAGG
2539-2	GCTCTAGACTGGGCGGATGCCAGTGC
2539-3	GCTCTAGAGCAGGCATCTCGCTGTTCC
2539-4	CCCAAGCTTAGGCCCTCGAAGTTCTGC
2540-1	CGGAATTCTTTGAACCGCCCCAAGTTGCTGC
2540-2	GCTCTAGATTCGGTGGTCTGGGCGAAGC
2540-3	GCTCTAGACTGCGCAACCAGCTCCTGTTCG
2540-4	CCCAAGCTTTTGTCGGCTTCCTCGGGCTG
2538-1	CGGAATTCTACTCGGCGAAGGCCTGCATCAGG
2538-2	GCTCTAGACGGACGTTCGGCGAGAACAAC
2538-3	GCTCTAGACATGAGGGCGGTTCGCACGAAC
2538-4	CCCAAGCTTGTAGCCGTCGAAGTGGAGGATGG
AGMU-QT1	CACGAAGATCGTCCAGGAG
AGMU-QT2	GCCATCCTCCATGAGGTAC
AGLT-QT1	CGTCAGGCCTCTGAGAACC
AGLT-QT2	GGAGCACCTCCTGGTACAG
4868-QT1	GAAGACGACCTGCTGTGAAAG

4868-QT2	GAAACACAAGGTTCGCATC
4867-QT1	GCCGTTCCTCTGACACTC
4867-QT2	GAGACGGCCAATCTTGATG
4866-QT1	GTGCGCTTCGTTCGTTCTC
4866-QT2	CGATTTCATCGAACGTGAC
AGMO-QT1	CGCTCCTGTTGGGTGGCT
AGMO-QT2	GGAGTTGTTGTTGGCCGC
2539-QT3	CCTGTGTCTGGTGCCCGC
2539-QT4	GCATCCTGCGATGACTCGG
2540-QT3	CCACCGAAGAGGCGGAAG
2540-QT4	GGAAGACGTGGCCGGAG
2541-QT1	GTGGACGGCCCCCACCTA
2541-QT2	GGTGGGCTTCTTCTTGGG
4867-O1	GCTCTAGACGCCGTTCCTCTGACACTC
4867-O2	GGAATTCCTATTCGCCGGACTGCTTG
GMOA-O1	CCCAAGCTTGGACCTGGCGTCTGTGAC
GMOA-O2	GGAATTCTCCTCCTCGTCGCGAG
