# Supplementary material for: Emergence and Modular Evolution of a Novel Motility Machinery in Bacteria
Source: PLoS Genet. 2011 Sep 8;7(9):e1002268. doi: 10.1371/journal.pgen.1002268 (PMC3169522; doi:10.1371/journal.pgen.1002268)
Supplement: Table S5 — Plasmids. (PDF) [file pgen.1002268.s010.pdf]

Table S5. Plasmids

Name	Description	Source
pBJ114	Used to create deletions, *galK*, Km^R	[23]
pSWU30	Tet^R used to integrate genes ectopically at $Mx8_{att}$	L. Søgaard-Andersen
pBJAglZY	pBJ114 with a cassette allowing construction of the *aglZ-yfp* chimeric gene	[12]
pBJΩMxan_1922	pBJ114 with an insertion cassette for Mxan_1922	This work
pBJΩMxan_3374	pBJ114 with an insertion cassette for Mxan_3374	This work
pBJΩMxan_1327	pBJ114 with an insertion cassette for Mxan_1327	This work
pBJΔgltD	pBJ114 with a deletion cassette for *gltD*	This work
pBJΔgltE	pBJ114 with a deletion cassette for *gltE*	This work
pBJΔgltF	pBJ114 with a deletion cassette for *gltF*	This work
pBJΔgltG	pBJ114 with a deletion cassette for *gltG*	This work
pBJΔgltH	pBJ114 with a deletion cassette for *gltH*	This work
pBJΔgltC	pBJ114 with a deletion cassette for *gltC*	This work
pBJΔgltK	pBJ114 with a deletion cassette for *gltK*	This work
pBJΔgltB	pBJ114 with a deletion cassette for *gltB*	This work
pBJΔgltA	pBJ114 with a deletion cassette for *gltA*	This work
pBJΔpilA	pBJ114 with a deletion cassette for *pilA*	Laboratory stock
pBJΔaglQ	pBJ114 with a deletion cassette for *aglQ*	[13]
pSWU30gltG	pSWU30 allowing expression of *gltG* from its own promoter at $Mx8_{att}$	This work
pSWU30gltFC	pSWU30 allowing expression of *gltF-mCherry* from its own promoter at $Mx8_{att}$	This work
pKT25	Bacterial two-hybrid T25 donor plasmid	Euromedex
pUT18	Bacterial two-hybrid T18 donor plasmid	Euromedex
pUT18AglR	AglR-T18 fusion donor construct	This work
pKT25GltG	GltG-T25 fusion donor construct	This work
