# Supplementary material for: Emergence and Modular Evolution of a Novel Motility Machinery in Bacteria
Source: PLoS Genet. 2011 Sep 8;7(9):e1002268. doi: 10.1371/journal.pgen.1002268 (PMC3169522; doi:10.1371/journal.pgen.1002268)
Supplement: Table S6 — Myxococcus strains. (PDF) [file pgen.1002268.s011.pdf]

Table S6. *Myxococcus* strains

Strain	Construction	Source	Genotype
DZ2	Wild type	Laboratory collection	WT
TM156	DZ2 *pilA: :tet*	Laboratory collection	Ω*pilA*
TM267	DZ2 *frzE::Tn5 Ω231*	David Zusman	ΩfrzE
DZ4770	DZ2 Δ*aglZ*	[24]	Δ*aglZ*
TM140	DZ2 pBJΩMxan_1922	This work	ΩMxan_1922
TM141	DZ2 pBJΩMxan_3374	This work	ΩMxan_3374
TM643	DZ2 pBJΩMxan_1327	This work	ΩMxan_1327
TM142	DZ2 Δ*gltD* (pBJΔgltD)	This work	Δ*gltD*
TM148	DZ2 Δ*gltE* (pBJΔgltE)	This work	Δ*gltE*
TM136	DZ2 Δ*gltF* (pBJΔgltF)	This work	Δ*gltF*
TM135	DZ2 Δ*gltG* (pBJΔgltG)	This work	Δ*gltG*
TM149	DZ2 Δ*gltH* (pBJΔgltH)	This work	Δ*gltH*
TM456	DZ2 Δ*gltC* (pBJΔgltC)	This work	Δ*gltC*
TM600	DZ2 Δ*gltK* (pBJΔgltK)	This work	Δ*gltK*
TM603	DZ2 Δ*gltB* (pBJΔgltB)	This work	Δ*gltB*
TM606	DZ2 Δ*gltA* (pBJΔgltA)	This work	Δ*gltA*
TM253	TM142 *pilA: :tet*	This work	Δ*gltD* Ω*pilA*
TM297	TM148 *pilA: :tet*	This work	Δ*gltE* Ω*pilA*
TM246	TM136 *pilA: :tet*	This work	Δ*gltF* Ω*pilA*
TM245	TM135 *pilA: :tet*	This work	Δ*gltG* Ω*pilA*
TM244	TM149 *pilA: :tet*	This work	Δ*gltH* Ω*pilA*
TM466	TM456 *pilA: :tet*	This work	Δ*gltC* Ω*pilA*
TM602	TM600 *pilA: :tet*	This work	Δ*gltK* Ω*pilA*
TM605	TM603 *pilA: :tet*	This work	Δ*gltB* Ω*pilA*
TM608	TM606 *pilA: :tet*	This work	Δ*gltA* Ω*pilA*
TM274	TM142 *frzE::Tn5 Ω231*	This work	Δ*gltD* ΩfrzE
TM275	TM148 *frzE::Tn5 Ω231*	This work	Δ*gltE* ΩfrzE
TM276	TM136 *frzE::Tn5 Ω231*	This work	Δ*gltF* ΩfrzE
TM277	TM135 *frzE::Tn5 Ω231*	This work	Δ*gltG* ΩfrzE
TM293	TM149 *frzE::Tn5 Ω231*	This work	Δ*gltH* ΩfrzE
TM467	TM456 *frzE::Tn5 Ω231*	This work	Δ*gltC* ΩfrzE
TM601	TM600 *frzE::Tn5 Ω231*	This work	Δ*gltK* ΩfrzE
TM604	TM603 *frzE::Tn5 Ω231*	This work	Δ*gltB* ΩfrzE
TM607	TM606 *frzE::Tn5 Ω231*	This work	Δ*gltA* ΩfrzE
TM330	DZ4770 *frzE::Tn5 Ω231*	This work	Δ*aglZ* ΩfrzE
TM392	TM330 ΔpilA (pBJΔpilA)	This work	Δ*aglZ* Δ*pilA* ΩfrzE
TM445	TM274 ΔpilA (pBJΔpilA)	This work	Δ*gltD* Δ*pilA* ΩfrzE
TM248	TM135 *mx8*$_{att}$*::gltG* (pSWU30gltG)	This work	Δ*gltG gltG*
TM273	TM136 *mx8*$_{att}$*::gltF-mCherry* (pSWU30gltFC)	This work	Δ*gltF gltF-mCherry*
TM406	TM273 ΔpilA (pBJΔpilA)	This work	Δ*gltF* Δ*pilA gltC-mCherry*
TM247	*agmU-mcherry*	[18]	*agmU-mcherry*
TM410	TM247 *pilA: :tet*	This work	Ω*pilA agmU-mcherry*
TM472	TM410 *aglZ-yfp* (pBJAglZY)	This work	Ω*pilA agmU-mcherry aglZ-yfp*
TM470	TM410 Δ*aglQ* (pBJΔaglQ)	This work	Δ*aglQ* Ω*pilA agmU-mcherry*
TM471	TM406 Δ*aglQ* (pBJΔaglQ)	This work	Δ*aglQ* Δ*gltF* Δ*pilA gltF-mCherry*
