# Supplementary material for: Emergence and Modular Evolution of a Novel Motility Machinery in Bacteria
Source: PLoS Genet. 2011 Sep 8;7(9):e1002268. doi: 10.1371/journal.pgen.1002268 (PMC3169522; doi:10.1371/journal.pgen.1002268)
Supplement: Table S7 — Plasmid constructions. (PDF) [file pgen.1002268.s012.pdf]

Table S7. Plasmid constructions

| Plasmid | Construction scheme[a] |
|---|---|
| pBJΩ1922 | A 1Kb internal fragment of Mxan_1922 was amplified from the DZ2 chromosome with primers 1922-1 / 1922-2 and cloned at the *EcoRI* and *Hind*III sites of pBJ114. |
| pBJΩ3374 | A 1Kb internal fragment of Mxan_3374 was amplified from the DZ2 chromosome with primers 3374-1 / 3374-2 and cloned at the *EcoRI* and *Hind*III sites of pBJ114. |
| pBJΩ1327 | A 750bp internal fragment of Mxan_1327 was amplified from the DZ2 chromosome with primers ins1327-1 / ins1327-2 and cloned at the *HindIII* and *BamHI* sites of pBJ114. |
| pBJΔgltD | Primer pairs DAgmU7 / DAgmU8 and DAgmU9 / DAgmU10 were used to amplify 1kb fragment upstream and downstream from the *gltD* open-reading frame. The upstream fragment was first cloned at the *EcoRI* and *KpnI* sites of pBJ114. Then, the downstream fragment was ligated at the *KpnI* and *HindIII* sites of the pBJ114-upstream fragment. |
| pBJΔgltE | Primer pairs DAglT1 / DAglT5 and DAglT6 / DAglT4 were used to amplify 1kb fragment upstream and downstream from the *gltE* open-reading frame. The upstream fragment was first cloned at the *EcoRI* and *BamHI* sites of pBJ114. Then, the downstream fragment was ligated at the *BamHI* and *HindIII* sites of the pBJ114-upstream fragment. |
| pBJΔgltF | Primer pairs D4868-1 / D4868-5 and D4868-6 / D4868-4 were used to amplify 1kb fragment upstream and downstream from the *gltF* open-reading frame. The upstream fragment was first cloned at the *EcoRI* and *BamHI* sites of pBJ114. Then, the downstream fragment was ligated at the *BamHI* and *HindIII* sites of the pBJ114-upstream fragment. |
| pBJΔgltG | Primer pairs D4867-1 / D4867-5 and D4867-6 / D4867-4 were used to amplify 1kb fragment upstream and downstream from the *gltG* open-reading frame. The upstream fragment was first cloned at the *EcoRI* and *BamHI* sites of pBJ114. Then, the downstream fragment was ligated at the *BamHI* and *HindIII* sites of the pBJ114-upstream fragment. |
| pBJΔgltH | Primer pairs D4866-1 / D4866-2 and D4866-3 / D4866-4 were used to amplify 1kb fragment upstream and downstream from the *gltH* open-reading frame. The fragments were then fused by overlap PCR and cloned at the *EcoRI* and *BamHI* sites of pBJ114. |
| pBJΔgltC | Primer pairs D2541-1 / D2541-2 and D2541-3 / D2541-4 were used to amplify 1kb fragment upstream and downstream from the *gltC* open-reading frame. The upstream fragment was first cloned at the *EcoRI* and *BamHI* sites of pBJ114. Then, the downstream fragment was ligated at the *BamHI* and *HindIII* sites of the pBJ114-upstream fragment. |
| pSWU30gltG | A fragment encompassing *gltG* and the *glt*G promoter region was amplified from the DZ2 chromosome with primers prom4867-BamHI / 4867EcoRI-4 and cloned at the *BamHI* and *EcoRI* sites of pSWU30. |
| pSWU30gltFC | Primers prom4868-1 / 4868-sansSTOP-2 were used to amplify the fragment *gltF* and its promoter from the DZ2 chromosome. Primers 4868-mcherry-3 / mcherry-4 were used to amplify the fragment *mCherry* from a plasmid containing the *mcherry* gene. Both fragments were fused by SOE-PCR and cloned at the *BamHI* and *HindIII* sites of pSWU30. |
| pBJΔgltA | Primer pairs 2540-1 / 2540-2 and 2540-3 / 2540-4 were used to amplify 1kb fragment upstream and downstream from the *gltA* open-reading frame. The upstream (*EcoRI* / *XbaI*) and downstream (*XbaI* / *HindIII*) fragment was cloned in one step at the *EcoRI* and *HindIII* sites of pBJ114. |
| pBJΔgltB | Primer pairs 2539-1 / 2539-2 and 2539-3 / 2539-4 were used to amplify 1kb fragment upstream and downstream from the *gltB* open-reading frame. The upstream (*EcoRI* / *XbaI*) and downstream (*XbaI* / *HindIII*) fragment was cloned in one step at the *EcoRI* and *HindIII* sites of |

| | |
|---|---|
| | pBJ114. |
| pBJΔgltK | Primer pairs 2538-1 / 2538-2 and 2538-3 / 2538-4 were used to amplify 1kb fragment upstream and downstream from the *gltK* open-reading frame. The upstream (*EcoRI* / *XbaI*) and downstream (*XbaI* / *HindIII*) fragment was cloned in one step at the *EcoRI* and *HindIII* sites of pBJ114. |
| pUT18AglR | A fragment encompassing *aglR* was amplified from the DZ2 chromosome with primers GMOA-O1 / GMOA-O2 and cloned at the *HindIII* and *EcoRI* sites of pUT18. |
| pKT25GltG | A fragment encompassing *gltG* was amplified from the DZ2 chromosome with primers 4867-O1 / 4867-O2 and cloned at the *XbaI* and *EcoRI* sites of pKT25. |

[a] All plasmid inserts were sequenced to ensure the absence of PCR-introduced mutations.
