# Supplementary material for: Emergence and Modular Evolution of a Novel Motility Machinery in Bacteria
Source: PLoS Genet. 2011 Sep 8;7(9):e1002268. doi: 10.1371/journal.pgen.1002268 (PMC3169522; doi:10.1371/journal.pgen.1002268)
Supplement: Text S1 — MXAN4864 and MXAN4865 may not be actual motility genes. (DOCX) [file pgen.1002268.s013.docx]

**Text S1**

**MXAN4864 and MXAN4865 may not be actual motility genes**

The predicted MXAN_4864 and MXAN_4865 overlap and are transcribed in opposite direction (not shown). In fact, the MXAN_4864 and MXAN_4865 ORFs are predicted with equal probability based on third position GC skew (data not shown); however, they both encode hypothetical proteins unique to *M. xanthus* and its very close relative *Stigmatella aurantiaca* DW4/3-1. Thus, these ORFs are likely false positives (MXAN_4865 is no longer annotated in the *M. xanthus* DK1622 genome) and we favour the hypothesis that the *agmV* transposon insertion (inserted in this region) did not disrupt an actual gene but exerted polar effects on the downstream *agmK* [1], potentially by disrupting critical promoter elements.
