# Supplementary material for: Emergence and Modular Evolution of a Novel Motility Machinery in Bacteria
Source: PLoS Genet. 2011 Sep 8;7(9):e1002268. doi: 10.1371/journal.pgen.1002268 (PMC3169522; doi:10.1371/journal.pgen.1002268)
Supplement: Text S2 — Justification for a glt nomenclature of the gliding motility machinery genes. (DOCX) [file pgen.1002268.s014.docx]

**Text S2**

**Justification for a *glt* nomenclature of the gliding motility machinery genes.**

We propose a new Glt nomenclature for the G1 and G2 genes. The evidence indicates that the G1 and G2 genes encode a trans-envelope complex that interacts with the AglRQS motor and transduces its activity to the cell surface. In consequence we rename those genes **gl**iding **t**ransducer genes (*glt*). This new nomenclature will be helpful for several reasons:

- There is currently no established nomenclature about A-motility genes in *Myxococcus*. Until now names have been given based on genetic screens and authors have used their own nomenclature to name the genes. Early genetic studies named gliding motility genes *agl* and *cgl*, depending on whether the motility defect can be rescued upon cell-cell contact or not [2,3]. More recent genetic screens introduced *agm* (**a**dventurous **g**liding **m**otility), *agn* (**a**dventurous **g**lidi**n**g) and *pgl* (**p**artial **gl**iding) [1,4]. Some genes are even named after two distinct nomenclatures even if they are transcribed in the same operon for example, *agmU*, *aglT* and *pglI* (we rename these genes *gltD*, *gltE* and *gltG*).

-The lack of a clear nomenclature also creates confusion when previously unknown motility genes must be named. For example, this study and also the study by Nan et al. [5] identify new motility functions for open reading frames such as MXAN4868, 4866, 2539 and 2540 that were previously uncharacterized. Many gene letters are already used by the various nomenclatures. The G2 cluster contains AgmO, MXAN2539, MXAN2540 and AgnA. In this example, not only it is not clear which of the *agm* or *agn* nomenclature should be used but *in fine* none is possible because AgmP, AgmQ or AgnB, AgnC are already used for other motility genes [1,4].

- Most of the genes names were given from loss of function transposon-based genetic screens. While clearly some of the genes encode the A-motility machinery, many other genes may encode unrelated functions. Presumably, general cellular defects, for example metabolic or cell division division defects, would also create gliding motility phenotypes. Thus, a Glt nomenclature inspired from flagellar genes (*fla*) or pilus genes (*pil*) would greatly clarify specific gene functions. Such approach has been used recently for the *nfs* system to name the G3 cluster genes with a function in sporulation [6]. For consistency, the *glt* genes were lettered according to their *nfs* homologues. For example *nfsA* is homologous to *gltA*, *nfsB* to *gltB* and so forth.
