# Supplementary material for: Emergence and Modular Evolution of a Novel Motility Machinery in Bacteria
Source: PLoS Genet. 2011 Sep 8;7(9):e1002268. doi: 10.1371/journal.pgen.1002268 (PMC3169522; doi:10.1371/journal.pgen.1002268)
Supplement: Text S3 — Principle of the phylogenomic analysis. (DOCX) [file pgen.1002268.s015.docx]

**Text S3**

**Principle of the phylogenomic analysis**

The phylogenomic analysis of a cellular system or complex starts with the comparison of the evolutionary history of its components (i.e. their phylogeny) to the phylogeny of the organisms carrying homologues of the corresponding genes [7,8]. This allows identifying: (i) the evolutionary origins of the components (i.e. in which lineage each of the components appeared), (ii) the events that changed their evolutionary histories (i.e. duplications, losses, transfers, fusions and splits of genes, gains or losses of functional domains, etc.) and (iii), the genomic events that have affected organization of the genes in genomes (i.e. gene cluster formation, disruption, etc.). At this step, working with complete genomic sequences is essential because it allows conclusive inference on the presence/absence of a gene in a given organism. Integrating information on each individual component of a system may thus reveal the evolutionary history of this particular system or complex. In an attempt to do so, we applied such approach to the genes of the G1, G2 and M1 clusters.
